# Insights into the Classification of Myasthenia Gravis

**DOI:** 10.1371/journal.pone.0106757

**Published:** 2014-09-05

**Authors:** Tetsuya Akaishi, Takuhiro Yamaguchi, Yasushi Suzuki, Yuriko Nagane, Shigeaki Suzuki, Hiroyuki Murai, Tomihiro Imai, Masakatsu Motomura, Kazuo Fujihara, Masashi Aoki, Kimiaki Utsugisawa

**Affiliations:** 1 Department of Neurology, Tohoku University Graduate School of Medicine, Sendai, Japan; 2 Division of Biostatistics, Tohoku University Graduate School of Medicine, Sendai, Japan; 3 Department of Neurology, Sendai Medical Center, Sendai, Japan; 4 Department of Neurology, Hanamaki General Hospital, Hanamaki, Japan; 5 Department of Neurology, Keio University School of Medicine, Tokyo, Japan; 6 Department of Neurology, Neurological Institute, Graduate School of Medical Sciences, Kyushu University, Fukuoka, Japan; 7 School of Health Sciences, Sapporo Medical University, Sapporo, Japan; 8 Medical Engineering Course, Department of Engineering, The Faculty of Engineering, Nagasaki Institute of Applied Science, Nagasaki, Japan; Research Inst. of Environmental Med., Nagoya Univ., Japan

## Abstract

**Background and Purpose:**

Myasthenia gravis (MG) is often categorized into thymoma-associated MG, early-onset MG with onset age <50 years, and late-onset MG with onset age ≥50 years. However, the boundary age of 50 years old between early- and late-onset MG remains controversial, and each category contains further subtypes. We attempted to classify MG from a statistical perspective.

**Methods:**

We analyzed 640 consecutive MG patients using two-step cluster analysis with clinical variables and discrimination analysis, using onset age as a variable.

**Results:**

Two-step cluster analyses categorized MG patients into the following five subtypes: ocular MG; MG with thymic hyperplasia (THMG); generalized anti-acetylcholine receptor antibody (AChR-Ab)-negative MG; thymoma-associated MG; and generalized AChR-Ab-positive (SP) MG without thymic abnormalities. Among these 5 subtypes, THMG showed a distribution of onset age skewed toward a younger age (p<0.01), whereas ocular MG and SPMG without thymic abnormalities showed onset age skewed toward an older age (p<0.001 and p<0.0001, respectively). The other 2 subtypes showed normal distributions. THMG appeared as the main component of early-onset MG, and ocular MG and SPMG without thymic abnormalities as the main components of late-onset MG. Discrimination analyses between THMG and ocular MG and/or SPMG without thymic abnormalities demonstrated a boundary age of 45 years old.

**Conclusions:**

From a statistical perspective, the boundary age between early- and late-onset MG is about 45 years old.

## Introduction

Myasthenia gravis (MG) is an autoimmune disease mediated by autoantibodies against molecules in the neuromuscular junction (NMJ), such as anti-acetylcholine receptor antibody (AChR-Ab) or anti-muscle-specific receptor tyrosine kinase antibody (MuSK-Ab) [1]. Each of these autoantibodies leads to distinct clinical characteristics [1]. Other concurrent striational autoantibodies also affect clinical features [2]. MG is often classified as follows based on the thymic abnormalities present and age at onset: thymoma-associated MG (TAMG); early-onset MG with age at onset <50 years; and late-onset MG with age at onset ≥50 years [3]–[5]. However, the use of 50 years as the boundary for age at onset remains controversial. “MG with thymic hyperplasia” (THMG), “sero-negative” (without AChR-Ab) and “double-seronegative” (with neither AChR-Ab nor MuSK-Ab) MG are also employed as subtypes in clinical settings. Furthermore, ocular MG represents a unique category distinguished from the generalized form [4]. The present study attempted to clarify subtypes of MG from a statistical perspective using two-step cluster analysis and discrimination analysis.

## Methods

### Patients

Among 676 consecutive MG patients surveyed in the Japan MG registry study of 2012 [4], [6], 640 adult patients for whom all the information required for the present analysis was available provided written informed consent [6] and participated in the present statistical study.

### Clinical factors

The following clinical factors were used as variables: sex; age at onset; disease duration; presence of thymoma; presence of thymic hyperplasia; positivity for AChR-Ab or MuSK-Ab; positivities for other concurrent autoantibodies (see below); MG Foundation of America (MGFA) clinical classification [7]; and MGFA post-intervention status (MGFA-PIS) as the current outcome [7]. The term thymic hyperplasia was assigned if the germinal center was observed in the thymus on histopathological examination, regardless of number, for non-thymomatous patients who underwent thymectomy [4]. Other concurrent autoantibodies analyzed were anti-ryanodine receptor antibodies (RyR-Ab), anti-nuclear antibodies, anti-SSA/Ro antibodies, anti-thyroglobulin/thyroperoxidase antibodies, thyroid-stimulating antibodies and rheumatoid factor.

All study protocols were approved by the ethics committee of Tohoku University School of Medicine, the ethics committee of Sendai Medical Center, the ethics committee of Hanamaki General Hospital, the ethics committee of Keio University School of Medicine, the ethics committee of Kyushu University School of Medicine, the ethics committee of Sapporo Medical University, the ethics committee of Nagasaki Institute of Applied Science, the ethics committee of Saitama Medical Center, the ethics committee of Toho University Medical Center Oh-hashi Hospital, the ethics committee of Tokyo Medical University or the ethics committee of Nagasaki Kawatana Medical Center. These clinical investigations have been conducted according to the principles expressed in the Declaration of Helsinki. Written informed consent was obtained from all patients prior to participation in the study.

### Statistical analysis

The regular cluster analysis divides subjects into classes simply according to distances (e.g. Euclidean distances) among variables, which may not be fitted for analysis simultaneously of both categorical and continuous variables with various levels of measurement and scale. On the other hand, two-step cluster analysis estimates log-likelihood and measures probability distribution of each variable, which is more suitable for the present clinical analysis.

Therefore, to classify the patients, we conducted two-step cluster analysis using SPSS Statistics Base 22 software (IBM, Armonk, New York, USA), which can extract clusters with high accuracy [8]. The number of clusters was automatically set by the statistical software to achieve the highest accuracy. Similarities of clusters were assessed with distance measures using log-likelihood distance. Clustering was achieved by a clustering feature tree based on an agglomerative clustering algorithm. Selection of optimal clustering was achieved using Schwarz's Bayesian criterion. After randomly separating the dataset into three equal-sized subsamples, results of this analysis were reconfirmed by 3-fold cross-validation.

Correlations between clinical factors were evaluated using the Spearman rank correlation. The boundary age between early- and late-onset MG was determined by discrimination analysis. Differences between groups were evaluated using the Mann-Whitney U test for continuous variables and the χ^2^ test for categorical variables. Values of *p*<0.05 were considered statistically significant.

## Results

### MG subtypes via two-step cluster analysis

First, two-step cluster analysis for the whole dataset (n = 640, see Table S1) suggested separation into the following two clusters: ocular MG (n = 143) and others. The quality of this cluster, as estimated using the interpretation model by Rousseeuw [9], was indicated as “fair” to “good” (reasonable evidence of cluster structure). Second, two-step cluster analysis was performed for the data excluding ocular MG, and suggested separations of THMG (n = 100) and generalized AChR-Ab-negative MG (n = 90) with “fair” cluster quality (reasonable evidence of cluster structure). Third, analysis of subjects after excluding ocular MG, THMG, and generalized AChR-Ab-negative MG suggested further separation of TAMG (n = 128) from residual generalized AChR-Ab-positive MG (SPMG) without thymic abnormalities (n = 179) with “fair” cluster quality. We completed the analysis at this step, because the quality of further clustering was “poor” if SPMG without thymic abnormalities underwent further analysis.

These results were reconfirmed by 3-fold cross-validation, which showed almost the same results in all three cluster analysis with subsamples.

### Characteristics of MG subtypes separated by cluster analysis

Clinical characteristics of each cluster are summarized in Table 1. Frequency histograms for onset age in generalized AChR-Ab-negative MG (Fig. S1) and TAMG (Fig. S2) showed normal distributions (p>0.05, p>0.05; Kolmogorov-Smirnov test for normality) with peaks around 35–39 years and 50–54 years, respectively. However, onset age of THMG showed a distribution skewed toward younger age (p<0.01, Kolmogorov-Smirnov test) with a peak around 25–29 years (Fig. S3). Conversely, frequency histograms for onset ages in ocular MG (Fig. S4) and SPMG without thymic abnormalities (Fig. S5) showed skewed distributions toward older age (p<0.001 and p<0.0001, respectively, Kolmogorov-Smirnov test) with peaks around 60–64 years and with peaks around 65–69 years (or an aging–dependent manner), respectively. Therefore, if adopted with these subtypes, early-onset MG appeared to be characterized by THMG, and late-onset MG by ocular MG and SPMG without thymic abnormalities.

**Table 1:** Clinical characteristics of each cluster of MG patients.

	Ocular MG n = 143	THMG n = 100	SNMG n = 90	TAMG n = 128	SPMG without thymic abnormalities n = 179	Total n = 640
Male (%)	44	20	22	37	37	35
Age at onset (years)	52.6±18.6	33.7±13.6	39.4±14.3	49.9±12.7	51.1±19.9	47.1±18.3
Duration of disease (years)	8.6±10.1	16.1±10.8	10.0±9.7	10.9±8.9	8.9±8.6	10.4±9.8
Thymoma (%)	12	0	0	100	0	23
Thymic hyperplasia (%)	4	100	0	0	0	17
AChR-Ab positivity (%)	78	90	0	100	100	79
MuSK-Ab positivity (%)	0	0	12	0	0	2
RyR-Ab positivity (%)	0	1	1	5	2	2
ANA positivity (%)	5	10	2	7	9	7
TG/TPO-Ab positivity (%)	10	13	3	6	11	9
MGFA classification (%)						
I	100	0	0	0	0	22.5
II	0	49.0	61.1	34.4	63.1	40.5
III	0	32.0	25.6	32.0	24.6	21.9
IV	0	10.0	4.4	10.9	5.6	5.9
V	0	9.0	8.9	22.7	6.7	9.2
Frequency of MM or better status (%)	64.3	45.0	32.2	44.5	55.9	50.9

**Footnotes**: AChR-Ab, anti-acetylcholine receptor antibodies; ANA, anti-nuclear antibodies; MG, myasthenia gravis; MGFA, MG Foundation of America; MM, minimal manifestations; MuSK-Ab, anti-muscle-specific receptor tyrosine kinase antibodies; RyR-Ab, anti-ryanodine receptor antibodies; SNMG, AChR-Ab-negative MG; SPMG, AChR-Ab-positive MG; TAMG, thymoma-associated MG; TG/TPO-Ab, anti-thyroglobulin/thyroperoxidase antibodies; THMG, MG with thymic hyperplasia.

RyR-Ab predominantly appeared in TAMG (Table 1). Positivities for other concurrent non-myasthenic autoantibodies were more frequent in THMG than in other subtypes (p<0.01), and less frequent in generalized AChR-Ab-negative MG than in other subtypes (p<0.0001) (Table 1). Details of other characteristics of TAMG, early- and late-onset MG for the present patients have been reported elsewhere [4], [6].

### Boundary age between early- and late-onset MG

**Figure 1:**
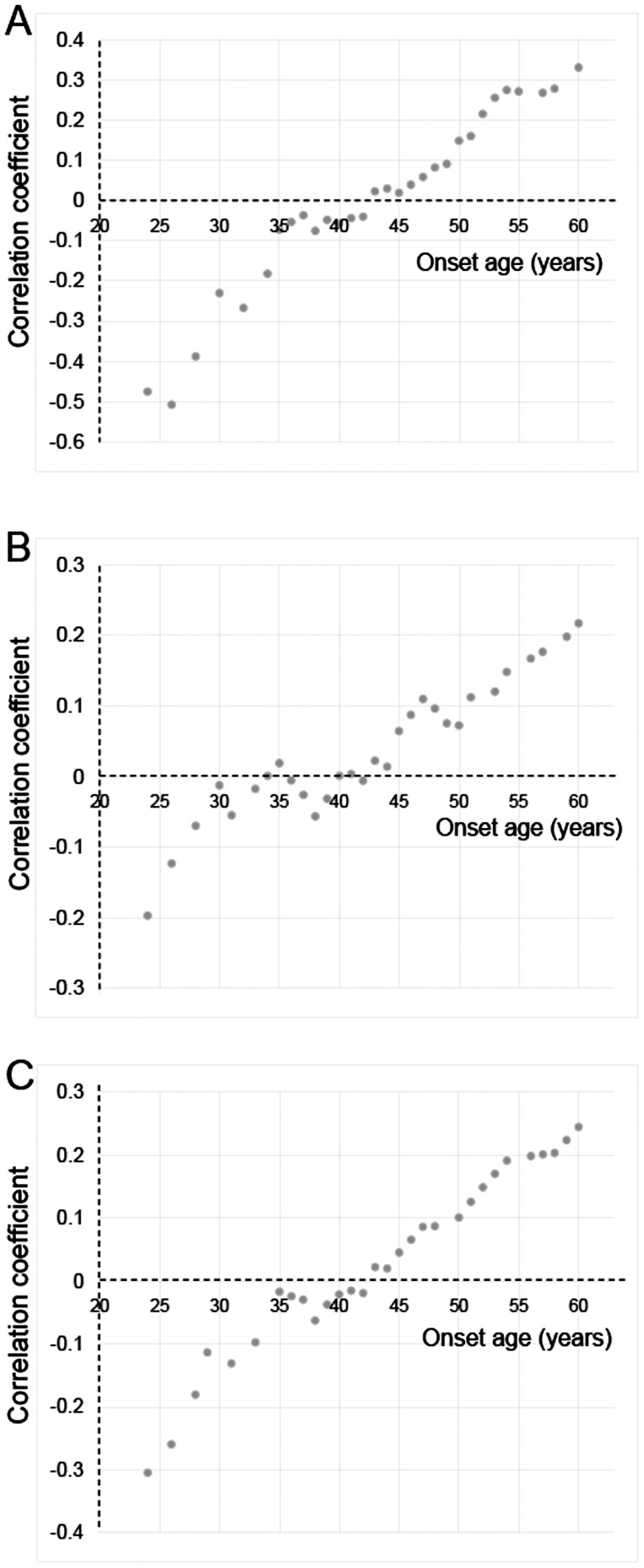
Changes in correlations as a function of age between patient frequency ratios of subtypes related to late-onset MG/early-onset MG + late-onset MG and onset age until a specific age. Ocular MG/ocular MG + THMG (A); SPMG without thymic abnormalities/SPMG without thymic abnormalities + THMG (B); and ocular MG + SPMG without thymic abnormalities/ocular MG + SPMG without thymic abnormalities + THMG (C).

We attempted to identify the boundary age between early- and late-onset MG using two statistical models. First, we analyzed changes in correlations as a function of age between frequency ratios of the subtypes related to late-onset MG/(early-onset MG + late-onset MG) and onset age until a specific age. Such changes in correlations are shown regarding ocular MG/(ocular MG + THMG) (Fig. 1-A), SPMG without thymic abnormalities/(SPMG without thymic abnormalities + THMG) (Fig. 1-B), and (ocular MG+ SPMG without thymic abnormalities)/(ocular MG + SPMG without thymic abnormalities + THMG) (Fig. 1-C). In these analyses, correlation coefficients between the frequencies of subtypes related to late-onset MG and onset age appeared to change from negative to positive at 45 years old (Fig. 1-A, B, C).

Second, we performed discrimination analysis with onset age as a variable to further establish the boundary age between early- and late-onset MG. The boundary age for discriminating THMG and SPMG without thymic abnormalities was calculated as 44.9 years old, and predictive values of the analysis were about 74%; these values were not particularly high, but were at the significant level for setting the boundary. In the same way, the boundary ages between THMG and ocular MG and between THMG and SPMG without thymic abnormalities + ocular MG were 44.8 and 47.5 years old (predictive values, 75% and 74%, respectively).

According to the findings from the two models, the boundary age between early- and late-onset MG was around 45 years old.

## Discussion

The present cluster analyses, without using early- and late-onset MG as variables, extracted THMG, ocular MG, and SPMG without thymic abnormalities as subtypes of MG. Distributions of onset age for THMG or ocular MG and SPMG without thymic abnormalities skewed toward younger or older ages. Given that ages at onset of the other two subtypes (generalized AChR-Ab-negative MG and TAMG) showed normal distributions and that TAMG is widely accepted as an independent unique subtype [4], [5], early- and late-onset MG were probably characterized by THMG and by ocular MG and SPMG without thymic abnormalities, respectively.

As clustering analyses are basically tools of exploratory data analysis for extracting data analogies, the present results need to be validated from the viewpoint of clinical utility and rationality. The separation of ocular MG with the strongest significance seems plausible, because patient backgrounds, clinical symptoms, and therapeutic responsiveness of ocular MG were totally different from those of other MG subtypes [4]. Another separated subtype THMG also is widely accepted as an independent and unique subtype [4], [5]. Furthermore, considering the separated cluster, TAMG may exhibit clinical characteristics more similar to SPMG without thymic abnormalities rather than to THMG, although both THMG and TAMG involve thymic pathology.

The boundary onset age between early- and late-onset MG was calculated to be around 45 years old by analyses between THMG and ocular MG and/or SPMG without thymic abnormalities, somewhat younger than but still relatively close to the often-used cut-off at 50 years old [4], [5]. The statistical perspective suggests that the boundary age should be set slightly younger than 50 years old. However, as thymectomy is usually not considered as a first-line treatment for patients classified with late-onset MG [1], estimating the boundary between early- and late-onset MG depending on the condition of the individual patient may be preferable.

In conclusion, MG was classified into ocular MG, THMG, generalized AChR-Ab-negative MG, thymoma-associated MG, and generalized SPMG without thymic abnormalities by two-step cluster analyses. THMG appeared to represent a component of early-onset MG, and ocular MG and SPMG without thymic abnormalities appear to represent components of late-onset MG. The boundary age between early- and late-onset MG was suggested as 45 years old. These results await external cross-validation with a different large-sized dataset in the future.

## Supporting Information

Figure S1 **Frequency histograms for onset age in generalized AChR-Ab-negative MG.**
(PDF)

Figure S2 **Frequency histograms for onset age in TAMG.**
(PDF)

Figure S3 **Frequency histograms for onset age in THMG.**
(PDF)

Figure S4 **Frequency histograms for onset age in ocular MG.**
(PDF)

Figure S5 **Frequency histograms for onset age in SPMG without thymic abnormalities.**
(PDF)

Table S1 **The whole dataset (n = 640) subjected to the present analysis.**
(PDF)
