# Supplementary figures and images for: Insights into the Classification of Myasthenia Gravis
Source: PLoS One. 2014 Sep 5;9(9):e106757. doi: 10.1371/journal.pone.0106757 (PMC4156422; doi:10.1371/journal.pone.0106757)

Figure S1

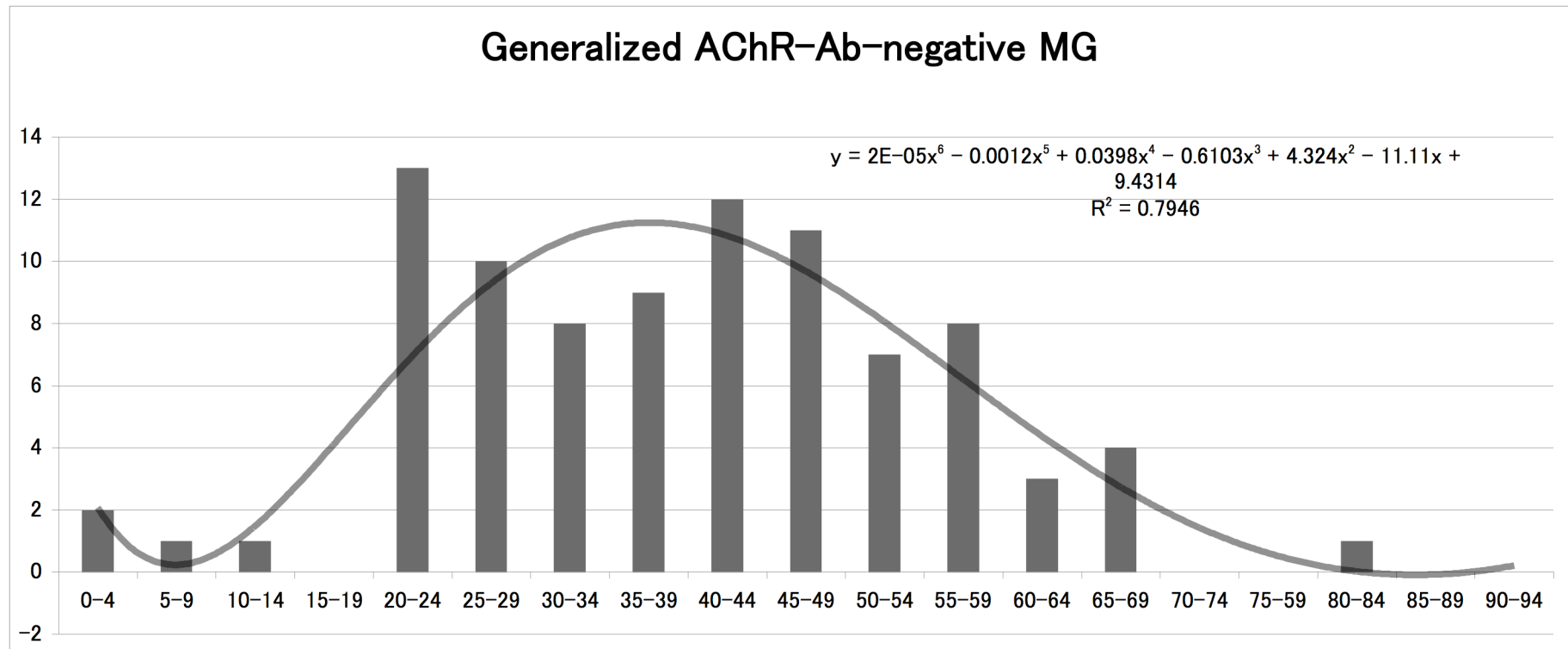

Supplement: Figure S1 — Frequency histograms for onset age in generalized AChR-Ab-negative MG. (PDF) [file pone.0106757.s001.pdf]

Figure S2

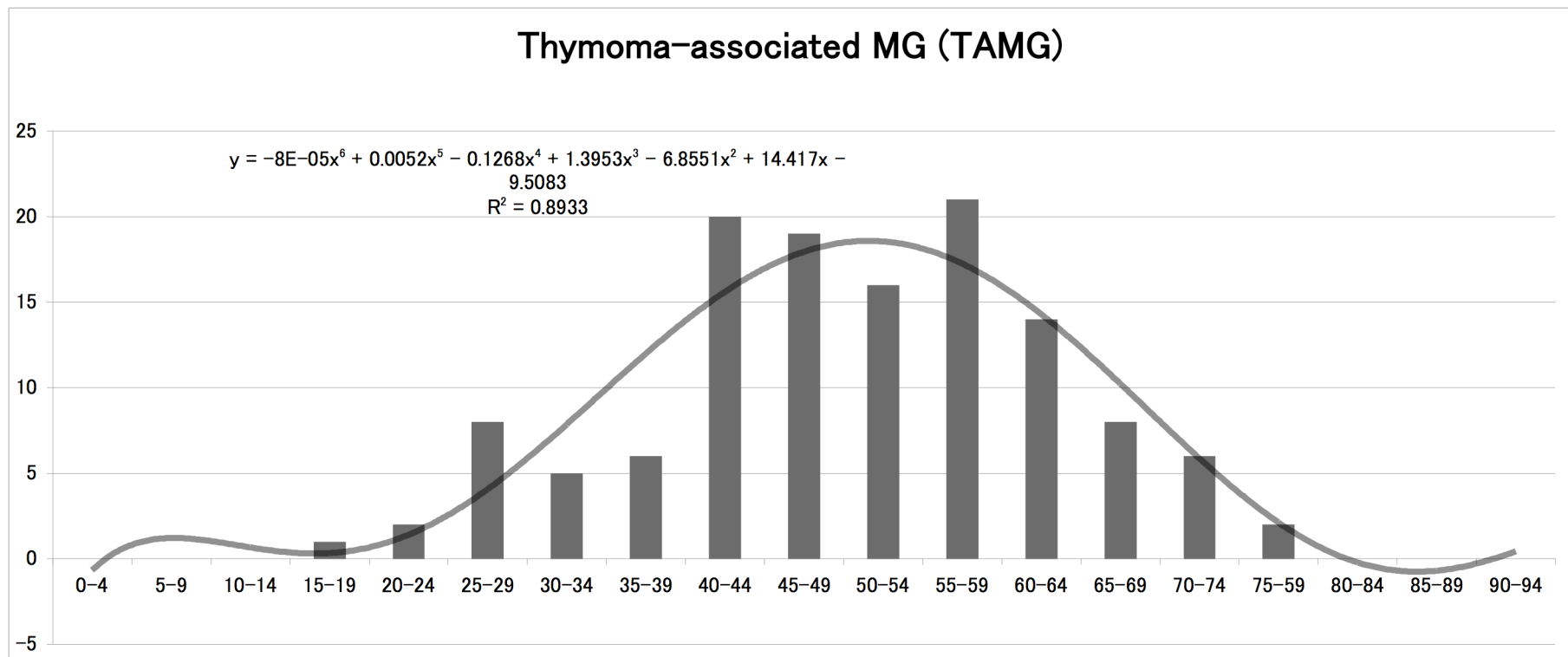

Supplement: Figure S2 — Frequency histograms for onset age in TAMG. (PDF) [file pone.0106757.s002.pdf]

Figure S3

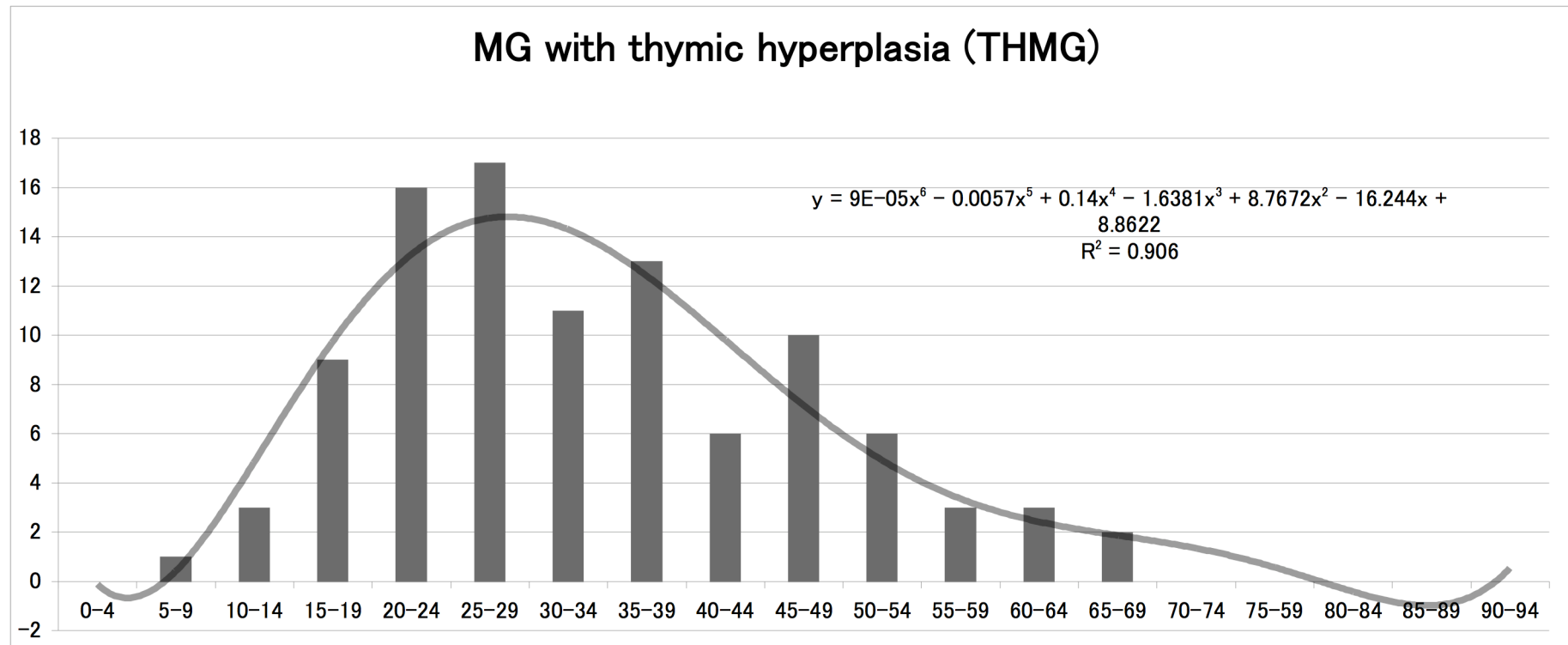

Supplement: Figure S3 — Frequency histograms for onset age in THMG. (PDF) [file pone.0106757.s003.pdf]

Figure S4

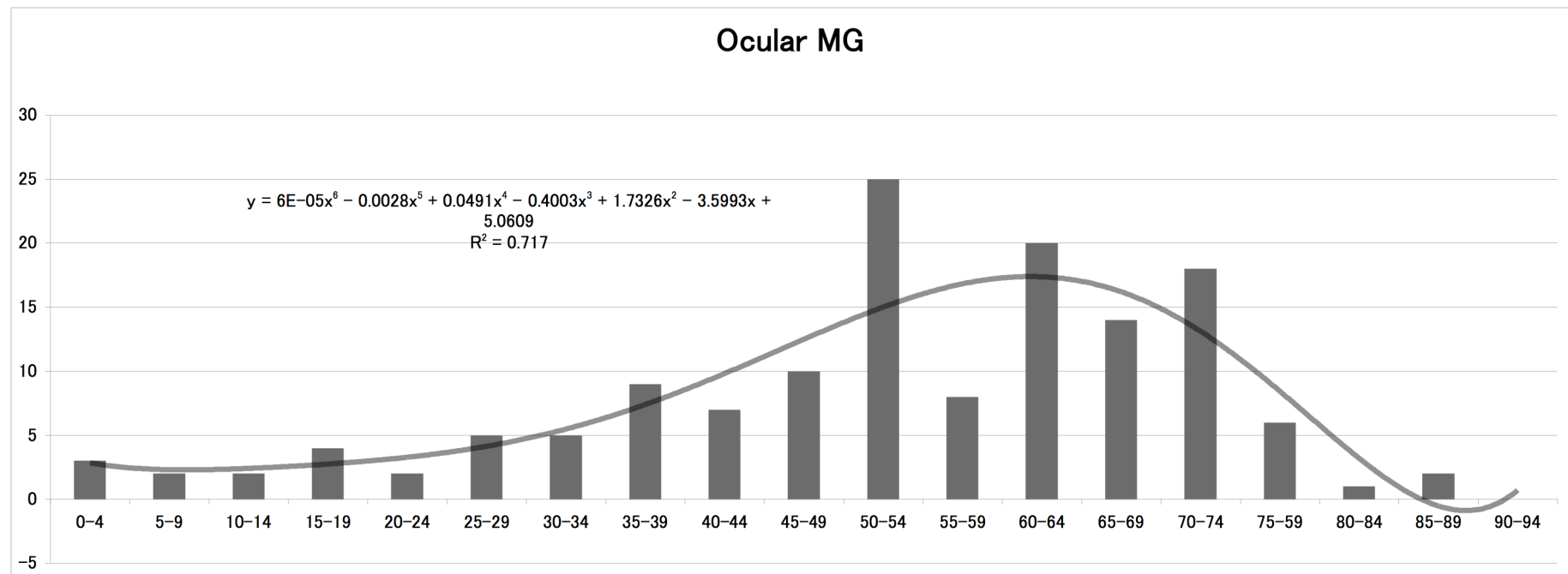

Supplement: Figure S4 — Frequency histograms for onset age in ocular MG. (PDF) [file pone.0106757.s004.pdf]

Figure S5

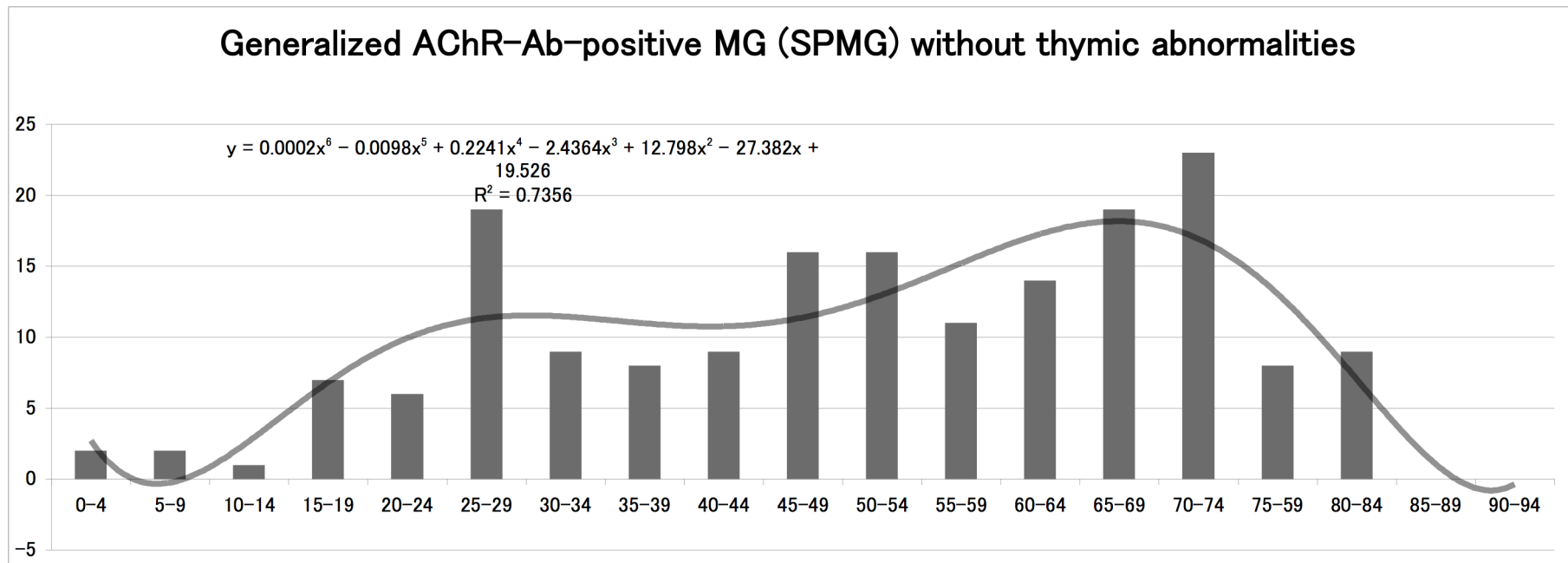

Supplement: Figure S5 — Frequency histograms for onset age in SPMG without thymic abnormalities. (PDF) [file pone.0106757.s005.pdf]
