# Supplementary material for: Insights into the Classification of Myasthenia Gravis
Source: PLoS One. 2014 Sep 5;9(9):e106757. doi: 10.1371/journal.pone.0106757 (PMC4156422; doi:10.1371/journal.pone.0106757)
Supplement: Table S1 — The whole dataset (n = 640) subjected to the present analysis. (PDF) [file pone.0106757.s006.pdf]

Table S1

	male	age	onset age	duration of disease
1	1	64	61	2.8
2	1	30	27	2.7
3	0	31	23	8
4	0	49.9	42	7.9
5	0	39	31	8.8
6	1	45	42	2.7
7	0	69	5	67
8	0	77	47	29.3
9	1	51	35	16.1
10	1	48	29	18.8
11	0	42	38	4.2
12	1	56.9	54.4	2.5
13	0	28	26	2
14	0	54.7	49.7	5
15	0	57	45	12
16	1	71	63	8
17	0	56	5	26
18	0	83	53	29.2
19	0	66	46	20.2
20	0	79	51	27.9
21	0	37	1	35.5
22	0	60	53	7
23	0	61	12	49
24	1	59	48	10.7
25	0	92	57	34.2
26	0	68	60	7.8
27	0	33	24	8.6
28	0	43	37	4.2
29	0	39	29	9
30	0	52	49	3
31	0	43	39	2.2
32	0	69	66	2.1
33	1	50	48	2
34	0	35	34	1.2
35	1	49	9	40
36	0	79	74	5
37	0	52	45	7
38	0	73	68	5
39	0	80	67	12
40	0	59	57	2
41	0	82	77	5
42	0	35	27	8
43	0	49.9	34	15.9
44	1	84.7	77.8	6.9
45	1	63.1	58.1	5
46	0	60	55	5
47	1	74.7	74.45	0.25
48	1	35	27	8
49	0	75	71	4
50	1	23	18	5
51	0	73	48	25
52	1	58	56	1.5
53	1	55	44	11
54	1	82	81	0.9

55	0	50	49	0.83
56	1	50	50	0.3
57	1	53	50	2.7
58	1	67	53	13.8
59	0	35	35	0.16
60	0	52	29	23
61	0	81	64	17.5
62	1	63	52	10.9
63	0	72	53	19
64	0	54	50	3.5
65	0	49	46	2.6
66	0	24	15	9
67	0	39	33	6.6
68	1	53	53	0.3
69	0	58	27	31
70	0	44	36	8
71	1	41	33	8
72	0	47	41	6
73	1	65	64	1.5
74	1	69	67	2
75	0	63	62	1.2
76	1	46	40	6
77	0	54	47	7
78	1	82	47	35
79	0	60	49	10.5
80	0	44	43	2
81	1	65	40	25
82	0	60	58	2
83	0	59	46	12.7
84	1	68	61	7
85	1	34	30	4
86	0	47	42	4.5
87	1	78	46	32.1
88	1	72	46	26.7
89	0	64	38	24.8
90	1	50	24	27.6
91	1	74	54	20.4
92	0	64	43	20.8
93	1	38	20	18.1
94	0	39	24	14.6
95	0	75	52	23.2
96	1	59	33	25.3
97	0	75	51	23.9
98	0	53	24	28.6
99	0	64	43	20.4
100	0	50	38	11.5
101	0	65	17	47.6
102	0	50	35	15
103	0	57	50	7
104	0	44	38	5.6
105	1	63	57	6.4
106	0	33	27	53
107	0	71	66	5.3
108	0	42	37	4.4
109	0	28	23	4.4
110	0	32	19	12
111	0	50	46	3.5
112	0	58	54	3.2

113	0	78	75	3.5
114	0	43	24	19
115	0	66	50	16.8
116	0	65	63	2.1
117	1	56	54	2
118	1	46	45	2
119	0	75	73	2
120	0	76	74	2
121	0	46	44	2.5
122	1	39	37	2
123	0	38	37	1.5
124	0	29	26	3.6
125	0	51	31	20
126	0	40	38	1.7
127	0	39	24	15
128	0	45	44	1.5
129	0	48	28	19
130	0	53	35	17
131	0	60	51	9
132	0	47	38	9
133	0	55	48	7
134	1	30	19	11
135	1	65	58	7
136	0	59	50	9
137	0	17	8	9
138	1	52	45	7
139	0	70	44	25
140	0	68	63	5
141	0	57	50	7
142	0	34	29	5
143	0	86	82	4
144	0	46	42	4
145	0	35	31	4
146	0	30	27	3
147	0	67	48	19
148	0	75	73	2.1
149	0	61	55	6
150	0	74	69	5
151	1	68	65	2.5
152	1	46	43	3
153	0	46	40	6
154	0	36	31	5
155	1	75.3	65.4	9.9
156	0	43.1	23.3	19.8
157	1	58.2	47.5	10.7
158	0	84.1	64.8	19.3
159	0	81.6	69.3	12.3
160	0	54.25	47	7.25
161	0	54.9	49	5.9
162	1	71.8	54.5	17.3
163	1	76.4	50	26.4
164	1	49.25	22	27.25
165	0	32.8	20	12.8
166	0	87.8	71.9	15.9
167	1	70.9	59	11.9
168	0	44.5	38.3	6.2
169	0	68.3	45	23.3
170	0	40.5	18.75	21.75

171	1	37.7	14	23.7
172	0	69.9	55.3	14.6
173	0	42.25	24.1	18.65
174	1	38.7	34.9	3.8
175	0	80.2	61.6	18.6
176	1	32.6	25	7.6
177	0	38.8	29	9.8
178	1	77.2	71.4	5.8
179	0	64	60.8	3.2
180	1	65.3	62.8	2.5
181	1	77.4	75.1	2.3
182	0	61.4	59.2	2.2
183	0	43.8	42.4	1.4
184	0	63.4	61.8	1.6
185	1	41.7	39.1	2.6
186	1	42.3	41.5	0.8
187	0	51	45	6
188	0	49	37	12
189	1	82	78	4
190	1	67	57	10
191	0	76	64	10
192	0	62	48	14
193	1	49	37	12
194	0	83	80	3
195	1	70	48	22
196	1	36	25	11.5
197	0	77	45	32
198	1	49	43	6
199	0	54	40	14
200	0	58	46	11.5
201	1	62	57	5.4
202	0	64	58	6.2
203	1	52	41	11.5
204	0	43	24	19.1
205	0	67	37	30.5
206	0	67	57	10
207	0	24	3	21
208	0	43	38	5
209	0	39	32	7
210	0	60	23	37
211	0	55	50	5.6
212	0	56	45	11.1
213	1	73	62	10.5
214	0	81	72	8.9
215	0	39	31	7.9
216	0	70	45	24.7
217	0	28	18	10.5
218	0	50	33	16.9
219	0	77	76	0.91
220	0	45	40	5.3
221	0	40	27	12.8
222	0	39	25	14.8
223	0	46	35	11.5
224	1	71	58	13
225	0	77	72	6
226	0	78	69	9.5
227	0	64	42	22
228	1	78	67	11.5

229	0	45	29	15.8
230	0	53	32	21
231	0	41	4	37
232	0	61	58	3
233	1	49	29	20
234	1	51	40	11
235	0	34	24	10
236	0	40	29	11
237	1	46	45	1.5
238	1	67	63	4
239	0	65	62	3.3
240	1	66	63	2.7
241	1	70	66	4.5
242	1	75	71	4.5
243	1	75	68	6.5
244	0	30	26	3.7
245	0	23	19	3.6
246	1	83	80	3.2
247	1	41	29	12.1
248	1	44	36	7.3
249	1	55	48	7.3
250	0	31	25	6.1
251	1	69	57	12.7
252	0	78	73	5.7
253	0	78	70	8.1
254	1	63	59	3.8
255	1	61	56	5.2
256	0	48	37	11.4
257	0	60	60	0.3
258	0	46	25	20.7
259	0	66	24	42
260	0	40	19	20
261	0	71	48	23
262	0	67	35	32
263	0	62	10	52
264	0	78	46	27
265	0	66	53	13
266	0	66	39	27
267	1	75	65	10
268	1	55	48	7
269	0	39	30	9
270	0	67	61	8
271	0	76	71	5
272	0	84	80	4
273	0	60	49	11
274	0	90	86	4
275	0	47	43	4
276	0	48	44	4
277	0	70	67	3
278	1	45	43	2.3
279	0	46	44	2.5
280	0	30	28	2
281	0	76	68	8
282	0	62	37	25
283	0	74	48	26
284	1	60	50	10
285	0	64	60	4
286	0	60	15	45

287	0	37	22	15
288	0	89	75	14
289	0	71	59	12.3
290	1	74	67	7
291	1	40	36	3.2
292	0	71	55	15.5
293	1	89	83	6
294	0	38	32	6
295	1	63	55	8
296	1	74	60	14
297	0	54	46	8
298	1	30	29	1.2
299	0	43	30	13
300	1	76	73	2.7
301	0	33	25	8
302	1	74	72	1.7
303	1	74	36	38
304	1	73	72	1
305	0	54	54	0.9
306	1	65	60	4.8
307	0	51	20	32
308	0	59	42	17
309	0	83	80	3
310	1	64	60	4.1
311	1	54	50	3.7
312	0	67	65	2.1
313	0	62	48	14.5
314	1	69	66	3.4
315	0	55	55	30.5
316	0	59	52	7.1
317	0	68	64	4
318	0	42	28	14.5
319	0	39	22	17.8
320	1	78	60	16.8
321	1	78	65	12.3
322	1	74	62	10.8
323	0	41	28	10.7
324	0	78	68	9.6
325	0	41	17	23.8
326	0	39	31	7.1
327	1	78	71	6.7
328	1	72	65	6.7
329	1	77	70	6.6
330	0	61	52	9.3
331	1	53	45	7.5
332	0	53	48	5.3
333	1	40	34	5.2
334	0	74	67	6.4
335	0	57	52	5.1
336	0	84	79	5
337	1	37	30	6.8
338	1	75	70	4.4
339	1	71	63	8
340	0	33	13	20
341	0	58	54	4.2
342	0	44	40	4.6
343	0	52	48	4.1
344	1	53	26	27

345	0	91	88	3.9
346	0	58	52	5.8
347	1	77	74	3.6
348	0	30	27	3.5
349	1	40	33	6
350	0	58	26	32
351	0	65	61	3.1
352	0	81	78	3.2
353	0	55	52	2.8
354	0	61	58	2.5
355	0	42	39	2.1
356	0	50	48	2.1
357	0	50	29	20
358	0	19	18	2
359	0	80	64	15
360	0	25	24	1.2
361	0	41	40	0.9
362	1	30	29	2
363	1	73	72	1
364	0	29	28	0.8
365	0	75	67	8
366	0	60	51	9
367	0	59	50	9
368	1	66	57	8
369	0	59	40	19
370	0	61	51	10
371	0	67	54	13
372	0	58	46	11
373	0	45	36	9
374	0	65	57	7
375	0	71	64	5
376	0	71	64	6
377	0	84	79	5
378	0	68	61	7
379	0	31	27	4.3
380	0	36	31	5
381	0	27	22	5
382	0	74	22	52
383	0	50	50	0.6
384	1	63	56	7
385	0	44	42	1.8
386	0	64	59	5
387	0	64.7	52	12.7
388	0	33.9	17	16.9
389	0	64.1	50.5	13.6
390	0	68.8	57.8	11
391	1	67	54.9	12.1
392	1	54.3	41.5	12.8
393	0	64.9	46	18.9
394	1	77.7	70.6	7.1
395	0	64.4	50.9	13.5
396	0	42.5	28.1	14.4
397	1	57.1	51.5	5.6
398	1	63.6	37.8	25.8
399	1	20.5	4	16.1
400	1	65	61	4
401	1	75.8	70.9	4.9
402	0	52.3	48.4	3.9

403	1	27.5	19.5	8
404	0	32.6	28.1	4.5
405	0	69.9	40	29.9
406	1	30.7	27.2	3.5
407	1	67.8	64.1	3.7
408	1	78.4	74.8	3.6
409	0	49.7	40.2	9.5
410	1	77.9	74.9	3
411	0	33.8	10	23.8
412	0	79.3	76.8	2.5
413	0	76.3	73.8	2.5
414	1	62.6	60.5	2.1
415	1	70.5	66.9	3.6
416	1	75.7	73.1	2.6
417	0	43.6	42.3	1.3
418	1	47.75	46.59	1.16
419	1	62.1	60	2.1
420	1	68.1	672	0.9
421	0	33.4	31.8	1.6
422	0	40	39.3	0.7
423	0	32	30.3	1.7
424	0	32.1	30.8	1.3
425	0	78.3	76.8	1.5
426	0	70.9	53	17.9
427	0	48.1	47.2	0.9
428	0	64	60	4
429	1	68	61	7
430	0	43	22	21
431	0	87	71	16
432	1	77	55	22
433	0	42	35	7
434	1	86	82	4
435	0	37	30	7
436	0	68	65	3
437	0	69	62	7
438	1	48	43	5
439	0	57	33	24
440	0	70	64	6
441	0	51	45	6
442	0	55	52	3
443	1	56	52	4
444	0	88	69	19
445	0	63	53	10
446	1	65	63	2
447	0	58	40	18
448	0	79	54	25
449	1	31	26	5
450	0	79	72	7
451	1	71	70	1.5
452	0	35	35	0.2
453	0	36	21	15
454	1	78	77	0.9
455	0	52	30	22
456	0	44	34	9.5
457	0	68	59	9.5
458	0	59	54	5.1
459	0	63	39	24
460	0	26	20	6

461	1	59	51	8
462	1	78	47	37
463	1	73	58	15
464	1	52	51	0.66
465	0	73	65	7.7
466	1	56	51	5.2
467	0	70	69	1.1
468	1	76	72	4
469	0	71	68	3.9
470	1	28	25	3
471	0	68	64	4
472	0	70	64	5.5
473	1	56	53	2.8
474	0	77	74	3.4
475	0	52	42	10.6
476	0	61	48	13
477	0	37	17	19.5
478	1	78	71	6.7
479	0	56	19	37
480	0	36	34	2.3
481	0	69	65	4.7
482	0	82	82	1
483	1	30	20	10
484	0	40	28	12.5
485	0	85	79	6.2
486	0	29	23	5.9
487	1	71	65	5.4
488	0	67	41	26.5
489	1	32	29	3.2
490	0	72	52	20
491	1	80	74	5.8
492	1	61	60	0.8
493	1	29	28	0.9
494	1	71	53	18.1
495	0	32	28	4.1
496	0	81	44	45
497	1	64	38	26
498	0	64	22	42
499	0	65	50	15
500	1	71	55	16
501	0	45	23	22
502	0	57	49	8
503	0	85	79	6
504	0	55	52	3
505	0	75	72	3
506	1	51	50	1.7
507	1	37	35	2
508	1	49	27	22
509	0	35	33	2
510	0	38	28	10
511	1	42	20	22
512	0	44	34	10
513	1	66	48	18
514	0	35	10	25
515	1	61	54	7
516	1	73	70	3
517	0	56	20	36
518	1	83	69	14

519	0	70	66	4.3
520	0	66	56	10
521	0	60	57	3.2
522	0	37	30	7.7
523	0	47	25	22.5
524	0	66	65	1.3
525	0	56	55	1
526	0	60	49	10.5
527	1	76	74	1.5
528	1	55	48	7.2
529	1	76	59	17.3
530	0	72	57	15
531	1	40	37	3
532	0	38	13	25
633	0	58	51	7.3
534	0	70	63	7.3
535	1	45	43	1.7
536	0	55	24	31
537	0	71	69	2.8
538	0	73	68	0.1
539	0	88	71	17
540	0	75	70	5
541	1	24	16	8
542	0	56	54	1.5
543	0	67	63	4.1
544	0	65	45	20
545	1	60	28	12.1
546	0	52	26	25.1
547	1	67	57	10.7
548	1	43	36	5.7
549	0	46	40	5
550	1	63	59	3.6
551	0	35	26	8.6
552	1	24	22	2.1
553	0	46	43	2.2
554	1	43	41	2.4
555	0	51	29	22
556	0	30	21	9
557	1	41	28	12
558	0	32	7	24
559	1	78	66	11
560	0	33	22	11
561	0	25	17	7
562	0	61	56	5
563	0	38	28	9
564	1	63	55	7
565	0	40	33	7
566	0	23	17	6
567	0	76	74	2
568	1	16	9	7
569	0	59	54	5
570	0	47	44	3
571	0	73	67	6
572	0	67	64	4.3
573	0	61	59	2
574	1	41	33	8
575	0	81.1	67.5	13.6
576	0	77.6	74.2	3.4

577	0	72.4	69.7	2.7
578	0	67	56	11
579	0	48	46	1.9
580	1	55	53	2
581	1	64	58	6
582	0	32	17	15
583	0	62	48	13
584	1	49	39	10
585	0	68	50	17
586	1	54	51	13
587	0	59	45	15
588	0	69	61	8.3
589	1	62	42	20
590	0	33	3	30
591	0	73	29	44
592	0	81	80	1
593	0	44	42	1.3
594	1	74	74	1
595	1	51	48	3
596	1	53	51	3
597	0	59	56	3
598	1	75	68	7
599	1	75	72	2.8
600	0	63	59	4
601	1	77	70	7
602	0	49	49	0.7
603	0	31	28	3.2
604	0	73	39	31.3
605	1	51	41	9.5
606	0	84	59	25
607	1	32	23	8.9
608	0	50	44	5.8
609	1	64	62	2.3
610	0	52	50	2.3
611	0	51	48	2.4
612	0	21	20	1.2
613	0	57	41	15
614	1	77	51	25
615	0	91	81	9
616	0	36	1	35
617	0	76	67	8
618	1	75	70	5
619	0	65	61	2.5
620	1	64.9	57.3	7.6
621	1	64.3	48.75	15.55
622	0	38.5	28.2	10.3
623	1	74.6	66.8	7.8
624	0	76.7	71	5.7
625	1	56.4	44	12.4
626	0	41.4	23.2	18.2
627	0	28.8	18.75	10.05
628	0	71.5	61.7	9.8
629	0	41.5	36.2	5.3
630	0	80.75	76.5	4.25
631	1	58.1	31.1	27
632	1	82	69	13
633	1	71	50	21
634	1	17	1	16

635	0	64	36	28
636	0	39	28	11
637	0	65	60	5
638	0	67	43	24
639	0	68	65	3
640	0	49	32	17

worst MGFA	crisis	AChR-Ab	MuSK-Ab	RyR-Ab
3	0	1	0	0
3	0	1	0	0
5	1	0	0	0
4	0	1	0	0
3	0	0	0	0
2	0	1	0	0
1	0	1	0	0
3	0	1	0	0
1	0	1	0	0
2	0	1	0	0
2	0	0	0	0
1	0	1	0	0
2	0	0	0	0
2	0	1	0	0
3	0	1	0	0
3	0	0	0	0
2	0	1	0	0
2	0	1	0	0
2	0	1	0	0
1	0	0	0	0
2	0	1	0	0
2	0	0	0	0
1	0	1	0	0
2	0	1	0	0
2	0	0	0	0
2	0	0	0	0
3	0	1	0	0
2	0	0	0	0
2	0	1	0	0
2	0	0	0	0
2	0	1	0	0
3	0	1	0	0
1	0	0	0	0
3	0	0	0	0
1	0	1	0	0
1	0	0	0	0
3	0	1	0	0
1	0	0	0	0
1	0	0	0	0
1	0	0	0	0
1	0	0	0	0
2	0	1	0	0
4	0	0	0	0
1	0	1	0	0
1	0	1	0	0
1	0	1	0	0
1	0	1	0	0
5	1	1	0	0
4	0	1	0	0
1	0	1	0	0
3	0	1	0	0
3	1	1	0	0
5	1	1	0	0
4	0	0	0	0

4	0	1	0	0
1	0	1	0	0
2	0	1	0	0
3	0	1	0	1
1	0	1	0	0
2	0	0	0	0
1	0	1	0	0
2	0	0	0	0
1	0	1	0	0
2	0	1	0	0
2	0	0	0	0
2	0	1	0	0
2	0	0	0	0
1	0	1	0	0
3	0	0	0	0
4	0	0	0	0
2	0	1	0	0
2	0	1	0	0
1	0	1	0	0
1	0	1	0	0
2	0	1	0	0
2	0	1	0	0
3	0	0	0	0
2	0	0	0	0
1	0	1	0	0
1	0	1	0	0
3	0	1	0	0
2	0	0	0	0
2	0	0	0	0
2	0	1	0	0
1	0	1	0	0
1	0	1	0	0
3	0	1	0	0
2	0	1	0	0
3	0	1	0	0
2	0	1	0	0
5	1	1	0	0
3	0	1	0	0
3	0	1	0	0
5	1	0	1	0
2	0	1	0	0
5	1	1	0	0
2	0	1	0	0
5	1	1	0	0
5	1	1	0	0
2	0	1	0	0
3	0	1	0	0
2	0	1	0	0
5	1	1	0	0
3	0	0	0	0
2	0	1	0	0
3	0	0	0	0
3	0	1	0	0
3	0	0	0	0
2	0	1	0	0
2	0	1	0	0
5	1	1	0	0
3	0	1	0	0

3	0	1	0	0
2	0	0	0	0
1	0	1	0	0
5	1	1	0	0
2	0	1	0	0
2	0	0	0	0
1	0	1	0	0
2	0	1	0	0
3	0	0	0	0
3	0	0	0	0
3	0	0	0	0
3	0	1	0	0
3	0	0	0	0
2	0	1	0	0
5	1	0	1	0
2	0	1	0	0
2	0	1	0	0
3	0	1	0	0
1	0	0	0	0
2	0	0	0	0
5	1	1	0	0
3	0	1	0	0
1	0	0	0	0
1	0	0	0	0
2	0	1	0	0
3	0	0	0	0
5	1	1	0	0
2	0	0	0	0
3	0	1	0	0
3	0	0	0	0
3	0	1	0	0
3	0	0	0	0
3	0	0	0	0
2	0	0	0	0
2	0	1	0	0
2	0	1	0	0
5	1	0	1	0
2	0	0	0	0
5	1	1	0	0
2	0	0	0	0
3	0	0	0	0
4	0	0	0	0
2	0	1	0	0
5	1	1	0	0
4	0	1	0	0
3	0	1	0	0
2	0	1	0	0
4	0	1	0	0
4	1	1	0	0
5	1	1	0	0
1	0	1	0	0
2	0	0	0	0
3	0	1	0	0
2	0	1	0	0
3	0	1	0	0
2	0	1	0	0
3	0	1	0	0
2	0	1	0	0

2	0	1	0	0
2	0	1	0	0
2	0	0	0	0
3	0	1	0	0
3	0	1	0	0
3	0	1	0	0
3	0	1	0	0
2	0	1	0	0
2	0	1	0	0
1	0	1	0	0
2	0	1	0	0
3	0	1	0	0
2	0	1	0	0
2	0	1	0	0
1	0	0	0	0
3	0	1	0	0
3	0	1	0	0
1	0	1	0	0
3	0	1	0	0
2	0	1	0	0
2	0	1	0	0
2	0	1	0	0
1	0	1	0	0
3	0	1	0	0
2	0	1	0	0
5	1	1	0	0
4	0	1	0	0
5	1	0	1	0
2	0	0	0	0
3	0	1	0	0
3	0	1	0	0
3	0	1	0	1
4	0	1	0	0
2	0	0	0	0
4	0	1	0	0
2	0	0	0	0
1	0	1	0	0
4	0	0	0	0
3	0	1	0	0
2	0	1	0	0
5	1	1	0	1
3	0	1	0	0
2	0	1	0	0
1	0	1	0	0
4	0	1	0	0
3	0	1	0	0
2	0	1	0	0
2	0	1	0	0
1	0	0	0	0
4	1	1	0	1
2	0	0	0	0
5	1	1	0	0
1	0	1	0	0
5	1	1	0	0
2	0	1	0	0
4	0	1	0	0
2	0	0	0	0
3	0	1	0	0

3	0	1	0	0
2	0	1	0	0
2	0	0	0	0
2	0	1	0	0
1	0	0	0	0
5	1	1	0	1
2	0	0	0	0
5	1	1	0	0
3	0	1	0	0
1	0	1	0	0
2	0	1	0	0
1	0	1	0	0
3	0	1	0	0
2	0	1	0	0
1	0	1	0	0
2	0	1	0	0
2	0	1	0	0
2	0	1	0	0
3	0	1	0	0
2	0	1	0	0
2	0	0	0	0
2	0	1	0	0
2	0	0	0	0
2	0	1	0	0
2	0	1	0	0
1	0	1	0	0
2	0	1	0	0
3	0	1	0	0
3	0	1	0	0
3	1	1	0	0
2	0	1	0	0
4	0	1	0	0
4	0	1	0	0
3	0	0	0	0
2	0	1	0	0
3	0	1	0	0
4	0	1	0	0
5	1	1	0	0
5	1	1	0	0
2	0	1	0	0
2	0	1	0	0
2	0	1	0	0
4	0	1	0	0
5	1	1	0	0
2	0	1	0	0
1	0	1	0	0
1	0	0	0	0
2	0	1	0	0
3	0	1	0	0
3	0	1	0	0
2	0	1	0	0
3	0	1	0	0
2	0	1	0	0
2	0	1	0	0
5	1	1	0	0
5	1	1	0	0
1	0	1	0	0
3	0	1	0	0

2	0	1	0	0
2	0	1	0	0
2	0	1	0	0
5	1	0	0	0
3	0	1	0	0
3	0	1	0	0
3	0	1	0	0
2	0	0	0	0
3	0	1	0	0
5	1	1	0	0
2	0	0	0	0
2	0	1	0	0
2	0	1	0	0
3	0	1	0	0
2	0	0	0	0
5	1	1	0	0
5	1	1	0	0
3	0	1	0	0
2	0	1	0	0
4	1	1	0	0
5	1	0	1	0
5	1	0	1	0
2	0	1	0	0
2	0	1	0	0
1	0	0	0	0
3	0	1	0	0
3	0	1	0	0
2	0	1	0	0
5	1	1	0	0
1	0	1	0	0
2	0	1	0	0
2	0	1	0	0
1	0	1	0	0
1	0	1	0	0
1	0	1	0	0
1	0	1	0	0
2	0	1	0	0
4	0	1	0	0
5	1	1	0	0
3	0	1	0	0
1	0	1	0	0
2	0	1	0	0
2	0	1	0	0
3	0	1	0	0
2	0	1	0	0
1	0	1	0	0
3	0	1	0	0
1	0	0	0	0
3	0	1	0	0
1	0	1	0	0
3	0	1	0	0
1	0	1	0	0
1	0	0	0	0
1	0	1	0	0
2	0	1	0	0
3	0	1	0	0
2	0	1	0	0
2	0	0	0	0

1	0	0	0	0
1	0	1	0	0
1	0	1	0	0
2	0	1	0	0
3	0	1	0	0
2	0	1	0	0
1	0	1	0	0
3	0	1	0	0
1	0	0	0	0
2	0	1	0	0
3	0	0	1	0
1	0	1	0	0
2	0	1	0	0
3	0	1	0	0
2	0	1	0	0
1	0	0	0	0
4	0	1	0	0
2	0	1	0	0
2	0	1	0	0
1	0	1	0	0
5	1	1	0	0
2	0	1	0	0
2	0	0	0	0
2	0	1	0	0
2	0	1	0	0
3	0	1	0	0
2	0	1	0	0
3	0	0	0	0
2	0	1	0	0
2	0	0	0	0
1	0	0	0	0
1	0	0	0	0
3	0	1	0	0
2	0	1	0	0
2	0	0	0	0
3	0	0	0	0
3	0	0	0	0
3	0	1	0	0
1	0	0	0	0
2	0	1	0	0
2	0	0	0	0
2	0	0	0	0
2	0	0	0	0
4	0	1	0	0
2	0	1	0	0
4	0	1	0	0
3	0	1	0	0
5	1	1	0	0
3	0	1	0	0
2	0	1	0	0
4	0	1	0	0
4	0	1	0	0
2	0	1	0	0
2	0	1	0	0
2	0	1	0	0
3	0	1	0	0
5	1	1	0	0
3	0	1	0	0

2	0	1	0	0
5	1	1	0	0
2	0	1	0	0
2	0	1	0	0
2	0	1	0	0
2	0	1	0	0
5	1	1	0	0
1	0	1	0	0
3	0	0	1	0
1	0	1	0	0
2	0	1	0	0
5	1	1	0	0
1	0	1	0	0
1	0	1	0	0
3	0	1	0	0
2	0	0	0	0
1	0	1	0	0
1	0	1	0	0
2	0	1	0	0
2	0	1	0	0
2	0	0	1	0
3	0	1	0	0
2	0	1	0	0
2	0	0	1	0
1	0	1	0	0
2	0	1	0	0
2	0	1	0	0
2	0	1	0	0
1	0	1	0	0
2	0	1	0	0
2	0	1	0	0
3	0	1	0	0
3	0	1	0	0
3	0	1	0	0
2	0	1	0	0
2	0	1	0	0
2	0	1	0	0
5	1	1	0	0
2	0	1	0	0
1	0	1	0	0
1	0	1	0	0
2	0	1	0	0
1	0	1	0	0
2	0	1	0	0
3	0	1	0	0
2	0	1	0	0
2	0	0	0	0
5	1	1	0	0
2	0	1	0	0
2	0	1	0	0
2	0	0	0	0
2	0	1	0	0
2	0	1	0	0
1	0	1	0	0
3	0	1	0	0
1	0	1	0	1
3	0	1	0	0
3	0	1	0	0

3	0	0	1	0
5	1	1	0	0
1	0	1	0	0
2	0	1	0	0
3	0	1	0	1
2	0	1	0	1
4	0	1	0	0
5	1	1	0	0
4	0	1	0	1
2	0	1	0	0
1	0	1	0	0
3	0	1	0	0
2	0	1	0	1
1	0	1	0	0
3	0	1	0	0
3	0	1	0	0
1	0	1	0	0
1	0	1	0	0
1	0	1	0	0
1	0	1	0	0
2	0	1	0	1
1	0	0	0	0
2	0	0	0	1
2	0	1	0	0
1	0	1	0	0
2	0	1	0	0
1	0	1	0	0
3	0	1	0	0
1	0	1	0	0
3	1	1	0	0
2	0	1	0	0
1	0	1	0	0
2	0	1	0	0
1	0	1	0	0
5	1	1	0	0
1	0	1	0	0
2	0	1	0	0
5	1	1	0	0
1	0	1	0	0
3	0	1	0	0
3	0	1	0	0
1	0	1	0	0
2	0	1	0	0
1	0	1	0	0
1	0	0	0	0
1	0	1	0	0
1	0	1	0	0
1	0	1	0	0
1	0	1	0	0
3	0	1	0	0
4	0	1	0	0
1	0	1	0	0
1	0	1	0	0
3	0	1	0	0
1	0	0	0	0
1	0	1	0	0
2	0	1	0	0
1	0	1	0	0

1	0	1	0	0
2	0	1	0	0
2	0	1	0	0
1	0	1	0	0
3	0	1	0	0
1	0	1	0	0
2	0	1	0	0
2	0	1	0	0
1	0	1	0	0
1	0	1	0	0
1	0	1	0	0
2	0	1	0	0
2	0	1	0	0
3	0	1	0	0
5	1	1	0	0
1	0	1	0	0
1	0	0	0	0
2	0	1	0	0
3	0	1	0	0
2	0	1	0	0
2	0	1	0	0
1	0	0	0	0
2	0	0	0	0
1	0	1	0	0
5	1	1	0	0
2	0	1	0	0
2	0	0	0	0
2	0	1	0	0
3	0	1	0	0
1	0	1	0	0
3	0	1	0	0
3	0	1	0	0
4	0	1	0	0
5	1	1	0	0
3	0	0	1	0
2	0	1	0	0
5	1	1	0	0
3	0	1	0	0
2	0	1	0	0
2	0	1	0	0
2	0	1	0	0
2	0	0	0	0
2	0	1	0	0
2	0	1	0	0
2	0	1	0	0
2	0	0	0	0
2	0	0	0	0
3	0	1	0	0
2	0	1	0	0
2	0	0	0	0
2	0	1	0	0
5	1	1	0	0
2	0	0	0	0
2	0	1	0	0
2	0	1	0	0
3	0	1	0	0
4	0	1	0	0
2	0	1	0	0

4	1	1	0	0
5	1	1	0	0
1	0	1	0	0
1	0	0	0	0
2	0	1	0	0
2	0	1	0	0
2	0	1	0	0
2	0	1	0	0
2	0	1	0	0
3	0	1	0	0
5	1	1	0	0
2	0	1	0	0
5	1	1	0	0
1	0	1	0	0
2	0	1	0	0
3	0	1	0	0
4	0	0	1	0
1	0	1	0	0
2	0	1	0	0
2	0	1	0	0
2	0	1	0	0
1	0	0	0	0
2	0	1	0	0
2	0	1	0	0
1	0	1	0	0
2	0	1	0	0
2	0	1	0	0
2	0	1	0	0
1	0	1	0	0
1	0	1	0	0
2	0	0	0	0
2	0	1	0	0
1	0	1	0	0
1	0	1	0	0
1	0	0	0	0
1	0	1	0	0
2	0	0	0	0
2	0	1	0	0
4	0	1	0	0
1	0	0	0	0
3	0	0	0	0
1	0	0	0	0
1	0	0	0	0
3	0	1	0	0
3	0	1	0	0
2	0	1	0	0
2	0	1	0	0
2	0	1	0	0
1	0	1	0	0
4	0	1	0	0
1	0	1	0	0
2	0	1	0	0
3	0	1	0	0
2	0	1	0	0
4	0	1	0	0
2	0	1	0	0
2	0	1	0	0
1	0	1	0	0

1	0	1	0	0
1	0	1	0	0
1	0	1	0	0
3	0	1	0	0
1	0	0	0	0
2	0	1	0	0

ANA	SS-A Ab	TG/TPO-Ab	TS-Ab	RF	thymectomy
0	0	0	0	0	1
0	0	0	0	0	0
0	0	0	0	0	0
1	0	0	0	0	1
0	0	0	0	0	1
0	0	0	0	0	0
0	0	0	0	0	0
0	0	0	0	0	0
0	0	0	0	0	1
0	0	0	0	0	1
0	0	0	0	0	0
0	1	1	0	0	1
0	0	1	0	0	0
0	0	0	1	0	1
0	0	0	0	0	1
0	0	0	0	0	0
0	0	0	0	0	1
0	0	0	0	0	1
0	0	0	0	0	0
0	0	0	0	0	1
0	0	0	0	0	0
0	0	0	0	0	0
0	0	0	0	0	0
0	0	0	0	0	0
0	0	0	0	0	0
0	0	0	0	0	0
0	0	0	0	0	1
0	0	0	0	0	0
0	0	0	0	0	0
0	0	0	0	0	0
0	0	0	0	0	0
0	0	0	0	0	0
0	0	0	0	0	0
0	0	0	0	0	0
0	0	0	0	0	0
0	0	0	0	0	0
0	0	0	0	0	0
0	0	0	0	1	0
0	0	0	0	0	0
0	0	0	0	0	0
0	0	0	0	0	0
0	0	0	0	0	0
0	0	0	0	0	1
0	0	0	0	1	0
1	0	1	0	0	0
0	0	0	0	0	0
0	0	0	0	1	0
0	0	0	0	0	1
0	0	1	0	0	0
0	0	1	1	0	0
0	0	0	0	0	1
0	0	0	0	0	0
0	0	0	0	0	1
0	0	0	0	0	0

0	0	0	0	0	1
0	0	0	0	0	0
0	0	0	0	0	1
0	0	0	0	0	1
0	0	0	0	0	0
0	0	0	0	0	0
0	0	0	0	0	0
0	0	0	0	0	0
0	0	0	0	0	0
0	0	1	0	0	1
0	0	0	0	0	0
0	0	0	0	0	1
0	0	0	0	0	0
0	0	1	0	0	0
0	0	0	0	0	0
0	0	0	1	0	1
0	0	1	0	0	1
0	0	0	0	0	1
0	0	0	0	0	0
0	0	0	0	0	0
0	0	1	0	0	0
0	0	0	0	0	0
0	0	0	0	0	0
0	0	0	0	0	0
0	0	0	0	0	1
0	0	0	0	0	0
0	0	0	0	0	1
0	0	0	0	0	0
0	0	0	0	0	1
0	0	0	0	0	1
0	0	0	1	0	1
1	0	0	0	0	0
0	0	0	0	0	1
0	0	0	0	0	1
0	0	0	0	0	1
0	0	0	0	0	1
0	0	0	0	0	1
0	0	0	0	0	1
0	0	0	0	0	1
0	0	0	0	0	1
0	0	0	0	0	1
0	0	0	0	0	1
0	0	0	0	0	1
0	0	0	0	0	1
0	0	0	0	0	1
0	0	0	0	0	1
0	0	0	0	0	1
0	0	0	0	0	1
0	0	0	0	0	1
0	0	0	0	0	0
0	0	0	0	0	0
0	0	0	0	0	0
0	0	0	0	0	1
0	0	0	0	0	0
0	0	0	0	0	1
0	0	0	0	0	1
0	0	0	0	0	1
0	0	0	0	0	0

0	0	0	0	0	0
0	0	0	0	0	1
0	0	0	0	0	0
0	0	0	0	0	0
0	0	0	0	0	0
0	0	0	0	0	0
0	0	0	0	0	0
0	0	0	0	0	0
0	0	0	0	0	0
0	0	0	0	0	0
0	0	0	0	0	0
0	0	0	0	0	0
0	0	0	0	0	1
0	0	0	0	0	1
0	0	0	0	0	0
0	0	0	0	0	0
0	0	0	0	0	1
1	0	0	0	1	1
0	0	0	0	0	0
0	0	0	0	0	1
0	0	0	0	0	1
0	0	0	0	0	0
0	0	0	0	0	0
0	0	0	0	0	0
0	0	0	0	0	0
0	0	0	0	0	0
0	0	0	0	0	1
0	0	0	0	0	0
0	0	1	1	0	0
0	0	0	0	0	0
0	0	0	0	0	0
0	0	0	0	0	0
0	0	0	0	0	0
0	0	0	0	0	0
0	0	0	0	0	1
1	0	1	0	0	0
0	0	0	0	0	0
0	0	0	0	0	0
0	0	0	0	0	1
0	0	0	0	0	0
0	0	0	0	0	0
0	0	0	0	0	1
0	0	0	0	0	0
0	0	0	0	0	1
1	0	0	0	1	1
0	0	0	0	0	1
0	0	0	0	0	0
1	0	0	0	0	1
1	0	0	0	1	1
1	0	0	0	0	1
0	0	0	0	0	0
0	0	0	0	0	0
1	0	0	0	0	1
1	0	0	0	0	0
0	0	0	0	0	1
0	0	0	0	0	1
1	0	1	0	0	1
1	1	0	0	0	1

1	1	0	0	0	1
0	0	0	0	0	1
0	0	1	0	0	1
0	0	0	0	0	1
1	0	1	0	0	1
1	0	1	1	0	1
0	0	0	0	0	1
0	0	0	0	0	0
0	0	0	0	0	0
0	0	1	1	0	1
0	0	0	0	0	0
0	0	0	0	1	0
0	0	0	0	0	1
1	0	0	0	0	1
0	0	0	0	0	0
0	0	0	0	0	1
0	0	0	0	0	0
0	0	0	0	0	0
0	0	0	0	0	0
0	0	0	0	0	1
0	0	1	0	0	0
0	0	0	0	0	1
0	0	0	0	0	1
0	0	0	0	0	0
0	0	0	0	0	1
0	0	0	0	0	1
0	0	0	0	0	1
0	0	0	0	0	0
0	0	0	0	0	0
0	0	0	0	0	1
0	0	0	0	0	1
0	0	0	0	0	1
0	0	0	0	0	1
0	0	0	0	0	1
0	0	0	0	0	1
0	0	0	0	0	0
1	0	0	0	0	0
0	0	0	0	0	0
0	0	0	0	0	1
0	0	0	0	0	1
0	0	0	0	0	1
0	0	0	0	0	1
0	0	0	0	0	1
0	0	0	0	0	1
0	0	1	0	0	1
0	0	0	0	0	1
0	0	0	1	0	1
0	0	0	0	0	1
0	0	0	0	0	0
0	0	0	0	0	1
0	0	0	0	0	1
0	0	0	0	0	1
0	0	0	0	0	0
0	0	0	0	0	1
0	0	0	0	0	1
0	0	0	0	0	1
0	0	0	0	0	1
0	0	0	0	0	1

0	0	0	0	0	1
0	0	0	0	0	1
0	0	0	0	0	0
0	0	0	0	0	1
0	0	0	0	0	1
0	0	0	0	0	1
0	0	0	0	0	0
0	0	0	0	0	1
0	0	0	0	0	1
0	0	0	0	0	0
0	0	0	0	0	0
0	0	0	0	0	0
0	0	0	0	0	1
0	0	0	0	0	0
0	0	0	0	0	0
0	0	0	0	0	0
1	0	0	0	0	0
0	0	0	0	0	0
0	0	0	0	0	1
0	0	0	0	0	1
0	0	0	0	0	0
0	0	0	0	1	1
0	0	0	0	0	0
0	0	1	0	0	1
0	0	1	0	0	1
1	0	0	0	0	0
0	0	0	0	0	1
1	1	0	0	1	1
0	0	1	0	0	0
0	0	0	0	0	1
0	0	0	0	0	1
0	0	0	0	0	1
0	0	0	0	0	1
0	0	1	0	0	1
0	0	1	0	0	1
0	0	1	0	0	1
0	0	0	0	0	1
0	0	0	0	0	1
0	0	0	0	0	1
0	0	0	0	0	1
0	0	0	0	0	1
0	0	0	0	0	1
0	0	0	0	0	1
0	0	0	0	0	1
0	0	1	0	0	1
0	0	0	0	0	0
0	0	0	0	0	0
0	0	1	0	0	1
0	0	0	0	0	1
0	0	0	0	0	1
0	0	0	0	0	1
0	0	0	0	0	1
1	0	1	0	0	0
0	0	0	0	0	1
0	0	0	0	0	1
0	0	0	0	0	1
0	0	0	0	0	1
0	0	0	0	0	1

0	0	0	0	0	1
0	0	0	0	0	0
0	0	0	0	0	1
0	0	0	0	0	0
0	0	0	0	0	1
0	0	0	0	0	1
0	0	0	0	0	0
0	0	0	0	0	0
0	0	0	0	0	1
0	0	0	0	0	1
0	0	0	0	0	0
0	0	0	0	0	0
1	0	1	0	0	1
0	0	0	0	0	0
0	0	0	0	0	0
0	0	0	0	0	0
0	0	0	0	0	1
0	0	0	0	0	0
0	0	0	0	0	1
0	0	0	0	0	0
0	0	0	0	0	1
0	0	0	0	0	0
0	0	0	0	0	0
0	0	0	0	0	1
0	0	0	0	0	0
0	0	0	0	0	1
0	0	0	0	0	1
0	0	0	0	0	1
0	0	0	0	0	1
0	0	0	0	0	1
0	0	0	0	0	1
0	0	0	0	0	1
0	0	0	0	0	0
0	0	0	0	0	0
0	0	0	0	0	0
0	0	0	0	0	0
0	0	0	0	0	1
0	0	0	0	0	0
0	0	0	0	0	1
0	0	0	0	0	1
0	0	0	0	0	0
0	0	0	0	0	0
0	0	0	0	0	0
0	0	0	0	0	1
0	0	0	0	0	1
0	0	0	0	0	1
0	0	0	0	0	1
0	0	0	0	0	0
0	0	0	0	0	1
0	0	0	0	0	0
0	0	0	0	0	1
0	0	0	0	0	0
0	0	0	0	0	0
0	0	0	0	0	0
0	0	0	0	0	1
0	0	0	0	0	1
0	0	0	0	0	0
0	0	0	0	0	1

0	0	0	0	0	0
0	0	0	0	0	0
0	0	0	0	0	0
0	0	0	0	0	1
0	0	0	0	0	1
0	0	0	0	0	1
0	0	0	0	0	0
0	0	0	0	0	0
0	0	0	0	0	0
0	0	0	0	0	1
0	0	0	0	0	0
0	0	0	0	0	1
0	0	0	0	0	1
0	0	0	0	0	1
0	0	0	0	0	0
0	0	0	0	0	0
0	0	0	0	0	1
0	0	0	0	0	0
0	0	0	0	0	0
0	0	0	0	0	1
1	1	0	0	0	1
0	0	0	0	0	0
0	0	1	0	0	1
0	0	1	0	0	1
0	0	0	0	0	0
0	0	0	0	0	0
0	0	0	0	0	0
0	0	0	0	0	0
0	0	0	0	0	0
0	0	0	0	0	0
0	0	0	0	0	0
0	0	0	0	0	0
1	0	1	0	0	0
0	0	0	0	0	0
0	0	0	0	0	0
0	0	0	1	0	1
1	1	0	0	0	0
0	0	0	0	0	0
0	0	0	0	0	0
0	0	0	0	0	1
0	0	0	0	0	0
0	0	0	0	0	0
0	0	0	0	0	1
0	0	0	0	0	1
0	0	0	0	0	0
0	0	0	0	0	1
0	0	0	0	0	1
1	0	0	0	0	1
0	0	0	0	0	1
0	1	0	0	0	0
0	0	0	0	0	1
0	1	0	0	0	1
0	0	1	0	0	1
0	0	0	0	0	1
0	0	0	0	0	0
0	0	1	0	0	1
0	0	0	0	0	0
0	0	0	0	0	1

0	0	0	0	0	1
0	0	1	0	0	0
0	0	0	0	0	0
0	0	0	0	0	1
0	0	0	0	0	0
0	0	0	0	0	0
0	0	1	0	0	1
0	0	0	1	0	0
1	0	0	0	0	0
0	0	1	0	0	0
0	0	0	0	0	0
1	1	1	0	0	1
1	0	1	0	0	0
0	0	0	0	0	0
0	0	0	0	0	1
0	0	0	0	0	0
0	1	0	0	0	0
0	0	1	0	0	0
1	0	0	0	0	1
0	0	0	0	0	0
0	0	0	0	0	0
0	0	0	0	0	0
0	0	0	0	0	0
0	0	1	0	0	0
0	0	0	0	0	0
0	0	0	0	0	1
0	0	0	0	0	1
0	0	0	0	0	1
0	0	0	0	0	1
0	0	0	0	0	1
0	0	0	0	0	1
0	0	0	0	0	0
0	0	0	0	0	1
0	0	0	0	0	1
0	0	0	0	0	0
0	0	0	0	0	0
0	0	0	0	0	1
0	0	1	0	0	1
0	0	1	1	0	1
0	0	0	0	0	0
0	0	0	0	0	0
0	0	0	0	0	0
0	0	0	0	0	1
0	0	0	0	0	0
0	0	0	0	0	1
0	0	0	0	0	1
0	0	0	0	0	0
1	0	1	0	0	0
0	0	0	0	0	0
0	0	0	0	0	0
0	0	0	0	0	0
0	0	0	0	1	0
0	0	0	0	1	1
0	0	0	1	0	0
0	0	0	0	0	1
0	0	1	0	0	1
0	0	0	0	0	1
0	0	0	0	0	1

0	0	0	0	0	0
0	0	1	0	0	1
0	0	0	0	0	0
0	0	1	0	0	1
0	0	0	0	0	1
0	0	0	0	0	1
0	0	0	0	0	1
0	0	0	0	0	1
0	0	0	0	0	1
0	0	0	1	0	1
0	0	1	0	0	1
0	0	0	0	0	1
0	0	0	0	0	1
0	0	0	0	0	1
0	0	0	0	0	1
0	0	0	0	0	1
0	0	0	0	0	1
0	0	0	0	0	1
0	0	0	1	0	0
0	0	0	0	0	1
0	0	0	0	0	1
0	0	0	0	0	0
0	0	0	0	0	0
0	0	1	0	0	0
0	0	0	0	0	0
1	0	0	0	0	1
0	0	0	0	0	0
0	0	0	0	0	1
0	0	0	0	0	0
0	0	0	0	0	1
1	0	0	0	0	1
0	0	1	0	0	0
0	0	0	0	0	0
0	0	0	0	0	0
0	0	0	0	0	1
0	0	0	0	0	0
0	0	0	0	0	1
0	0	0	0	0	1
0	0	0	0	0	0
0	0	0	0	0	1
0	0	0	0	0	1
0	0	0	0	0	0
0	0	1	0	0	0
0	0	0	0	0	1
0	0	0	0	0	0
0	0	0	0	0	0
0	0	0	0	0	0
0	0	0	0	0	0
0	0	1	1	0	1
0	0	0	0	0	1
0	0	0	0	0	1
0	0	0	0	0	0
0	0	0	0	0	0
0	0	0	0	0	0
0	0	0	0	0	0
0	0	0	0	0	0
0	0	0	0	0	1
0	0	0	0	0	1

0	0	0	0	0	0
0	0	0	0	0	0
0	0	0	0	0	1
0	0	0	0	0	1
0	0	0	0	0	1
0	0	0	0	0	0
0	0	0	0	0	1
0	0	0	0	0	1
0	0	0	0	0	0
0	0	0	0	0	1
0	0	0	0	0	1
0	0	0	0	0	1
0	0	0	0	0	1
0	0	0	0	0	1
0	0	0	0	0	1
0	0	0	0	0	1
0	0	0	0	0	0
0	0	0	0	0	0
0	0	0	0	0	0
0	0	0	0	0	0
0	0	0	0	0	1
0	0	0	0	0	0
0	0	0	0	0	1
0	0	0	0	0	0
0	0	0	0	0	1
0	0	0	0	0	0
0	0	0	0	0	1
0	0	0	0	0	1
0	0	0	0	0	1
0	0	0	0	0	0
0	0	0	0	0	1
0	0	0	0	0	1
0	0	0	0	0	1
0	0	0	0	0	1
0	0	0	0	0	0
0	0	0	0	0	0
0	0	0	0	0	1
0	0	0	0	0	0
0	0	0	0	0	1
0	0	0	0	0	0
0	0	0	0	0	1
0	0	0	0	0	0
0	0	0	0	0	0
0	0	0	0	0	1
0	0	0	0	0	1
0	0	0	0	0	0
0	0	0	0	0	0
0	0	0	0	0	1
0	0	0	0	0	0
0	0	0	0	0	0
0	0	0	0	0	0
0	0	0	0	0	1
0	0	0	0	0	1
0	0	0	0	0	0
1	0	0	0	0	1
0	0	0	0	0	1
1	0	0	0	0	1
0	0	0	0	0	0

1	1	1	0	0	0
1	1	1	0	0	1
1	0	0	0	0	1
0	0	0	0	0	0
0	1	0	0	0	1
0	0	1	1	0	1
0	0	0	0	0	1
0	0	0	0	0	1
0	0	0	0	0	1
0	0	0	0	0	1
0	0	0	0	0	1
0	0	0	0	0	1
0	0	0	0	0	1
0	0	0	0	0	0
0	0	0	0	0	0
0	0	1	0	0	0
0	0	0	0	0	0
0	0	0	0	0	0
0	0	0	0	0	0
0	0	0	0	0	1
0	0	0	0	0	1
0	0	0	0	0	0
0	0	0	0	0	0
0	0	0	0	0	0
0	0	0	0	0	0
0	0	0	0	0	1
0	0	0	0	0	1
0	0	0	0	0	1
0	0	0	0	0	0
0	0	0	0	0	1
0	0	0	0	0	1
0	0	0	0	0	0
0	0	0	0	0	0
0	0	0	0	0	1
0	0	0	0	0	0
0	0	0	0	0	1
0	0	0	0	0	0
0	0	0	0	0	1
0	0	0	0	0	0
0	0	0	0	0	0
0	0	0	0	0	0
0	0	0	0	0	0
0	0	0	0	0	0
0	0	0	0	0	1
1	0	0	0	0	1
0	0	1	1	0	1
0	0	1	0	0	0
1	0	0	0	0	1
0	0	0	1	0	1
1	0	0	0	0	1
1	0	1	0	0	1
0	0	0	0	0	0
0	0	0	0	0	1
0	0	0	0	0	1
0	0	0	0	0	1
0	0	0	0	0	1
0	0	0	0	0	1
0	0	0	0	0	0

0	0	0	0	0	0
0	0	1	0	0	0
0	0	0	0	0	0
0	0	0	0	0	0
0	0	0	0	0	1
1	0	1	0	0	0

thymoma	thymic hyperplasia	prednisolone peak dose	total QMG score
1	0	10	9
0	0	20	13
0	0	50	20
0	1	50	2
0	0	50	13
0	0	20	7
0	0	35	8
0	0	30	21
1	0	30	5
1	0	60	9
0	0	0	14
0	0	15	8
0	0	0	12
1	0	0	7
0	0	20	19
0	0	0	22
0	1	0	16
1	0	0	16
0	0	0	15
0	0	0	8
0	0	30	13
0	0	0	9
0	0	0	8
0	0	10	13
0	0	0	17
0	0	0	13
0	0	0	19
0	0	15	9
0	0	0	13
0	0	0	11
0	0	0	4
0	0	20	15
0	0	0	6
0	0	20	13
0	0	30	7
0	0	10	6
0	0	10	13
0	0	0	6
0	0	15	6
0	0	0	3
0	0	20	9
0	0	5	14
0	1	40	22
0	0	0	6
0	0	0	8
0	0	0	9
0	0	0	13
0	1	30	32
1	0	20	17
0	0	0	7
0	1	50	18
0	0	10	8
1	0	30	18
0	0	30	15

1	0	20	19
0	0	0	8
0	0	25	7
1	0	40	16
0	0	0	7
0	0	25	23
0	0	30	19
0	0	0	16
0	0	20	17
0	1	45	10
0	0	15	10
0	1	20	8
0	0	40	7
0	0	0	3
0	0	30	11
0	1	50	28
0	1	40	6
0	1	20	6
0	0	0	4
0	0	0	5
0	0	0	5
0	0	15	9
0	0	0	18
0	0	20	19
0	0	40	8
0	0	0	11
0	1	0	14
0	0	25	9
0	0	30	11
1	0	30	13
0	1	15	7
0	0	0	7
1	0	0	11
1	0	40	13
0	1	15	16
0	1	40	6
1	0	100	8
1	0	30	9
1	0	60	15
0	0	40	11
1	0	0	8
1	0	60	12
1	0	0	13
0	1	50	12
1	0	60	10
0	1	0	10
0	1	60	11
1	0	25	6
0	1	30	13
0	0	25	17
0	0	10	5
0	0	25	12
1	0	20	14
0	0	20	18
0	1	10	9
0	0	50	12
1	0	20	10
0	0	25	18

0	0	10	8
0	0	30	11
0	0	0	7
1	0	40	9
0	0	10	7
0	0	0	3
0	0	0	7
0	0	0	9
0	0	15	12
0	0	30	8
0	0	25	12
0	0	20	10
0	1	15	10
0	0	10	10
0	0	80	14
0	0	0	6
1	0	35	6
0	0	64	15
0	0	60	4
0	0	15	7
1	0	5	13
0	0	40	3
0	0	10	3
0	0	10	5
0	0	10	6
0	0	10	4
1	0	60	14
0	0	5	10
0	0	10	6
0	0	20	11
0	0	5	12
0	0	5	11
0	0	10	14
0	0	5	9
0	0	20	7
0	0	5	7
0	0	40	10
0	0	5	8
1	0	60	9
0	0	5	9
0	0	5	7
0	0	15	13
0	0	60	8
0	1	50	7
0	0	40	9
1	0	30	8
0	0	50	8
1	0	60	5
1	0	40	11
1	0	60	3
0	0	15	7
0	0	0	6
0	1	10	11
0	0	30	12
1	0	0	12
0	1	50	10
0	1	30	6
0	1	40	13

0	1	50	6
1	0	0	5
0	1	0	8
1	0	30	6
1	0	30	6
0	1	30	12
0	1	0	11
0	0	5	5
0	0	30	6
0	0	10	3
0	0	20	10
0	0	20	7
1	0	15	3
1	0	0	7
0	0	0	7
1	0	30	8
0	0	10	14
0	0	25	11
0	0	10	13
1	0	20	9
0	0	5	8
0	0	15	10
0	1	15	11
0	0	5	13
0	1		14
0	0	80	17
1	0	30	21
0	0	70	14
0	0	0	18
1	0	40	19
0	0	25	10
1	0	60	7
1	0	40	12
0	1	0	9
0	1		31
0	0	15	18
0	0	60	9
0	0	45	18
0	0	7.5	17
0	0	25	14
1	0	40	9
1	0	60	19
0	0	60	10
1	0	0	16
0	1	50	16
1	0	35	9
0	1	30	13
0	0	50	11
0	0	60	5
1	0	50	17
0	0	25	12
0	1	12.5	13
0	0	0	3
1	0	60	14
0	0	10	19
1	0	30	7
0	0	0	15
0	0	50	12

0	0	30	14
0	0	0	9
0	0		7
0	0	40	9
1	0	0	8
1	0	40	18
0	0	0	7
0	0	40	13
1	0	20	7
0	0	30	3
0	0	10	5
0	0	30	1
0	0	60	5
0	0	50	4
0	0	10	3
0	0	15	3
0	0	0	4
0	0	20	5
0	1	0	6
0	1	0	0
0	0	30	5
0	1	5	7
0	0	10	5
0	0	20	5
1	0	40	7
0	0	0	3
1	0	50	5
0	1	15	5
0	0	30	6
1	0	60	11
0	1	40	6
0	1	50	11
0	1	40	6
0	1	50	12
0	1	50	3
1	0	15	10
1	0	50	4
0	1	50	6
0	0	50	6
1	0	50	4
0	0	45	6
0	1	40	8
1	0	50	18
0	0	47.5	5
0	1	20	3
0	0	15	4
0	0	15	3
0	1	45	3
0	0	50	5
1	0	45	4
0	1	45	5
0	1	30	6
0	0	0	4
0	1	50	7
0	1	50	9
0	0	50	4
0	0	45	3
0	1	50	7

0	1	50	5
1	0	10	13
1	0	0	12
0	0	10	10
1	0	35	12
1	0	27.5	11
0	0	40	10
0	0	0	10
1	0	60	9
1	0	60	15
0	0	0	11
0	0	0	12
0	1	50	13
0	0	30	12
0	0	10	14
0	0	10	22
1	0		21
1	0	0	12
1	0	10	6
0	0	60	10
0	0	60	24
0	0		12
0	0	0	20
1	0	50	8
0	0	10	3
1	0	50	7
0	1	50	13
0	0	20	6
1	0	60	17
1	0	0	4
1	0	10	10
0	1	5	8
0	0	20	3
0	0	10	6
0	0	0	5
0	0	0	6
0	1	60	6
0	0	30	7
0	0	50	1
0	1	20	4
0	0	0	3
0	0	10	5
0	0	30	3
1	0	20	7
0	0	20	6
1	0	0	1
0	0	10	6
0	0	0	3
1	0	20	4
0	0	0	4
1	0	10	7
0	0	0	3
0	0	0	3
0	0	0	0
1	0	0	3
1	0	25	1
0	0	20	7
0	1	30	5

0	0	0	3
0	0	0	4
0	0	0	3
0	0	20	8
1	0	20	4
1	0	0	5
0	0	0	5
0	0	15	8
0	0	0	3
1	0	10	4
0	0	20	7
1	0	0	3
0	1	40	6
0	0	0	6
0	0	10	7
0	0	0	3
1	0	20	4
0	0	30	4
0	0	10	4
1	0	10	2
1	0	20	7
0	0	10	3
0	0	10	6
0	0	5	1
0	0	20	3
0	0	10	8
0	0	10	4
0	0	60	7
0	0	10	5
0	0	10	4
0	0	0	5
0	0	5	5
0	0	10	7
0	0	5	3
0	0	5	7
0	1	5	7
0	0	10	7
0	0	50	10
0	0	0	2
0	0	60	5
0	0	5	3
0	0	10	7
0	0	40	3
0	0	60	7
0	0	0	7
1	0	50	7
0	0	60	0
1	0	60	2
0	0	35	5
0	0	60	2
1	0	40	4
0	1	60	5
0	0	0	5
0	0	0	2
0	0	50	1
1	0	40	2
0	0	60	5
0	1	45	7

1	0	60	2
0	0	60	8
0	0	0	3
0	1	0	0
0	0	0	3
0	0	60	4
1	0	60	1
0	0	10	3
0	0	45	4
0	0	10	4
0	0	10	4
1	0	30	2
0	0	0	0
0	0	0	2
1	0	20	3
0	0	10	0
0	0	0	6
0	0	0	1
0	1	10	3
0	0	0	7
0	0	50	3
0	0	30	2
0	0	0	4
0	0	10	2
0	0	0	2
1	0	7.5	5
1	0	30	3
0	1	20	14
0	0	20	18
1	0	20	12
1	0	50	10
0	0	7.5	2
0	1	10	11
0	1	5	13
0	0	15	7
0	0	20	8
0	1	40	9
0	1	5	11
1	0	10	10
0	0	0	3
0	0	5	1
0	0	40	16
1	0	0	8
0	0	5	9
1	0	20	7
0	1	50	7
0	0	20	0
0	0	15	7
0	0	5	2
0	0	0	7
0	0	20	3
0	0	0	5
0	1	20	4
0	0	0	4
1	0	30	9
1	0	0	6
0	1	30	8
0	1	0	3

0	0	20	3
1	0	30	7
0	0	0	5
0	0	30	6
1	0	30	8
0	0	50	3
1	0	20	4
0	0	30	9
0	0	40	2
0	0	30	4
0	0	0	3
0	1	10	6
0	0	30	7
0	0	30	3
0	1	0	9
0	0	20	5
0	1	25	4
1	0	15	2
0	0	10	3
0	0	0	7
0	1	0	9
0	0	20	4
0	0	20	4
0	0	10	3
0	0	15	3
0	0	25	2
0	0	0	2
1	0	60	5
0	0	30	0
1	0		2
0	0	30	2
0	0	0	3
0	0	0	1
0	0	5	1
1	0	40	2
0	0	10	2
0	1	50	0
0	1	45	3
0	0	0	1
0	1	50	3
0	1	50	3
0	0	0	1
0	0	2.5	2
1	0	15	2
0	0	0	2
0	0	0	1
0	0	0	1
0	0	0	0
0	1	0	2
0	1	50	4
0	1	50	1
0	0	30	2
0	0	0	5
0	0	30	6
0	0	0	2
0	0	0	6
0	1	0	7
0	0	7.5	9

0	0	5	4
0	0	10	3
1	0	0	7
0	1	0	9
1	0	40	7
0	0	0	6
1	0	0	4
0	1	50	7
0	0	0	8
1	0	0	5
1	0	0	9
1	0	35	6
1	0	30	6
0	1	60	8
1	0	50	5
1	0	0	3
0	0	0	2
0	0	0	9
0	0	10	2
0	0	60	6
1	0	60	7
0	0	30	7
0	1	50	5
0	0	0	2
1	0	35	4
0	0	20	1
0	1	0	1
0	1	80	2
1	0	20	2
0	0	10	0
1	0	50	1
1	0	20	2
0	0	40	2
1	0	20	2
0	0	25	2
0	0	60	1
1	0	50	1
0	0	10	3
0	0	10	1
0	0	10	1
1	0	10	3
0	0	10	1
0	0	10	2
1	0	5	0
1	0	10	1
0	0	10	0
0	0	10	4
0	1	10	0
0	0	5	4
0	0	10	0
0	0	10	3
1	0	15	4
0	0	0	7
0	0	5	2
0	0	5	4
1	0	30	1
0	0	60	3
0	0	10	2

0	0	10	2
0	0	10	1
1	0	0	2
0	0	0	2
0	1	25	2
0	1	40	2
0	1	50	2
1	0	40	2
0	1	50	3
0	1	50	0
1	0	60	5
0	0	7.5	6
1	0	60	4
0	0	30	5
0	0	40	6
0	0	10	0
0	0	20	0
0	0	5	2
0	0	20	2
1	0	20	2
1	0	30	2
0	0	20	2
0	0	10	2
0	0	10	2
0	0	15	0
1	0	20	1
0	1	20	0
0	0	0	3
0	0	30	1
1	0	0	3
0	0	15	2
0	0	0	5
0	0	0	2
1	0	0	2
0	0	0	2
1	0	0	3
0	0	30	1
0	0	50	1
0	0	0	7
0	0	20	2
0	0	10	4
0	0	10	2
0	0	0	3
0	1	60	3
0	0	60	3
0	0	50	0
0	0	60	2
1	0	30	2
0	0	40	1
0	1	40	2
0	1	0	3
0	0	0	4
0	1	50	2
1	0	30	3
0	0	60	1
0	0	50	2
0	1	40	2
0	0	0	0

0	0	0	0
0	0	0	0
0	0	0	2
0	0	0	2
0	0	30	3
0	0	0	5

total MG composite	total MG QOL 15	total MG ADL
6	10	6
12	31	7
33	51	17
1	7	2
10	44	7
4	23	4
7	36	4
20	35	10
3	13	2
10	21	6
10	27	7
7	24	6
13	20	10
4	42	2
23	36	11
31	37	15
14	16	10
11	22	2
7	21	5
7	13	4
6	9	3
6	3	3
7	1	4
11	27	9
8	21	7
9	24	9
15	19	10
10	28	9
10	11	5
7	31	7
6	15	5
10	38	9
5	15	7
12	24	8
7	25	5
2	17	2
8	27	5
3	17	3
2	15	2
2	7	2
5	6	4
11	19	6
24	22	12
4	35	3
7	31	7
7	16	5
7	20	6
30	54	13
12	38	12
7	17	5
12	21	8
7	24	5
13	22	11
20	42	9

21	19	6
7	40	5
7	30	5
17	28	9
4	34	4
31	40	14
12	16	4
18	23	8
13	34	5
9	8	5
10	25	4
8	14	6
7	20	1
2	6	4
7	16	2
31	56	18
6	15	3
4	40	4
5	32	4
3	40	3
3	8	1
6	13	3
17	39	8
11	9	6
5	46	5
5	24	2
11	30	6
7	29	6
14	41	6
11	33	7
7	46	2
3	9	3
9	17	7
7	4	6
12	15	10
6	33	6
7	28	7
5	20	4
9	16	4
8	14	2
5	32	2
8	27	6
11	16	6
6	6	4
5	13	2
5	5	0
8	9	3
7	53	10
12	37	9
14	44	11
2	4	2
12	36	7
12	9	7
21	52	13
5	23	8
8	9	0
12	40	10
16	39	11

7	24	5
5	1	3
4	7	6
5	18	8
4	6	4
1	7	1
2	11	4
7	0	2
7	38	4
9	17	8
9	20	8
18	15	6
9	35	9
8	17	8
10	10	6
1	10	2
6	19	4
16	53	8
2	3	2
5	17	2
11	29	5
2	34	2
3	16	3
1	4	1
5	19	0
3	13	0
17	37	7
7	28	3
5	38	1
13	9	3
12	15	6
9	21	4
13	42	9
5	21	2
5	4	1
1	17	2
13	45	7
11	22	4
11	37	4
9	33	2
9	32	5
7	35	5
12	23	7
2	3	1
6	6	3
6	19	2
8	32	11
5	10	1
3	18	4
2	7	2
6	5	4
6	4	5
7	9	7
3	3	3
6	16	3
3	5	1
6	25	1
12	32	7

4	2	3
7	6	4
5	14	3
8	20	7
2	44	5
4	3	4
4	22	4
1	19	4
4	5	0
4	21	3
8	40	7
3	39	6
4	7	2
4	20	5
3	2	2
6	19	4
13	33	4
5	2	6
7	15	3
4	20	6
10	12	5
9	32	6
6	22	5
9	8	4
13	17	4
23	17	10
27	19	10
11	16	6
22	19	10
24	33	7
13	8	6
10	44	8
19	38	8
9	12	2
40	26	13
22	3	8
5	25	5
23	31	7
24	29	10
22	17	9
16	22	4
26	24	10
16	36	9
18	26	1
18	28	5
13	8	3
13	6	9
10	20	8
3	7	1
20	27	5
9	42	6
9	22	9
0	7	2
12	10	8
20	23	8
9	17	3
14	13	6
10	6	5

17	20	7
8	8	3
10	29	8
11	13	8
6	4	5
23	37	12
8	6	3
25	29	11
7	11	5
1	4	2
4	3	1
1	1	2
3	16	2
2	2	2
1	1	2
1	19	2
2	2	1
3	5	3
4	4	3
2	10	1
3	1	1
6	4	5
4	1	3
3	1	2
5	23	2
2	4	1
4	4	1
3	3	3
4	11	2
12	30	7
4	25	2
9	38	8
6	30	3
10	36	9
1	17	4
8	15	2
2	33	5
4	13	3
4	26	2
2	22	2
5	22	4
8	14	3
16	39	11
4	24	3
1	14	1
2	11	1
1	19	1
3	5	1
5	24	2
3	2	1
3	21	1
4	27	2
2	30	2
5	2	2
7	3	0
2	8	1
1	36	1
5	11	3

3	6	0
6	18	2
2	23	2
10	21	7
8	36	3
6	7	7
9	44	6
11	13	6
10	26	7
9	13	4
5	15	4
5	28	3
4	37	5
6	10	3
13	18	7
16	43	12
14		7
9	33	6
3	34	1
7	15	5
30	52	13
10	35	7
16	21	6
6	4	2
2	34	3
3	2	4
12	14	2
6	3	4
26	31	10
1	1	1
6	19	5
3	10	4
3	0	3
1	7	1
2	3	1
1	15	0
1	8	2
7	9	1
1	1	0
2	4	1
1	5	1
2	16	3
2	8	1
8	26	9
0	4	0
1	11	1
5	0	5
1	5	1
2	3	0
1	0	1
2	2	2
3	14	2
1	4	0
0	2	0
1	14	1
0	8	0
1	6	0
4	12	3

1	28	2
3	24	2
1	0	0
2	23	2
2	14	1
1	6	0
1	1	1
4	12	2
1	21	1
1	7	2
4	16	4
1	1	1
5	3	4
1	11	2
2	11	1
1	12	1
2	9	4
3	20	5
2	18	3
0	4	0
4	1	0
3	6	2
4	19	3
1	6	1
5	6	0
5	8	2
1	9	1
5	17	1
3	5	0
1	4	1
1	2	1
2	5	1
5	12	2
2	23	1
4	1	0
6	6	1
5	28	1
9	13	3
1	6	1
5	6	1
2	15	2
2	8	0
1	2	2
2	2	3
1	4	4
7	13	4
0	11	3
4	2	1
4	7	2
0	0	0
1	3	0
0	4	2
1	7	2
3	4	2
0	8	0
0	13	2
0	31	0
3	6	1

0	0	0
1	5	0
0	25	2
0	6	2
0	1	0
0	19	0
1	3	1
1	10	1
0	2	0
0	3	0
1	15	4
0	0	0
0	1	0
0	21	2
1	1	2
0	5	0
1	0	0
0	1	0
0	2	3
0	9	0
0	8	2
0	5	0
0	0	0
4	1	2
1	4	2
9	28	0
3	11	3
8	15	6
12	18	6
7	16	2
1	5	1
0	0	1
9	10	3
9	16	1
0	15	0
5	7	2
2	12	1
2	27	0
3	18	2
3	1	3
0	13	4
5	17	3
1	8	0
3	8	5
5	13	2
0	14	0
0	3	0
5	10	0
0	6	1
5		3
0	3	3
1	4	1
0	5	1
5	48	6
9	17	3
6	5	3
6	3	3
3	0	0

	2	8	2
	0	1	0
	2	4	2
	1	3	0
	11	0	1
	2	6	2
	3	23	2
	9	15	3
	0	0	0
	5	13	2
	0	11	3
	5	4	0
	4	10	1
	0	7	2
	10	17	4
	4	0	4
	0	1	1
	0	1	0
	0	6	0
	3	16	2
	11	13	5
	3	4	1
	1	1	1
	4	1	3
	1	0	1
	0	0	0
	0	1	1
	2	1	1
	0	1	0
	0	0	0
	0	4	0
	1	0	1
	0	2	0
	0	0	0
	0	34	0
	0	14	1
	0	15	1
	1	1	1
	0	16	1
	0	6	2
	1	6	2
	0	2	1
	0	0	0
	0	15	0
	0	3	1
	0	2	1
	1	8	1
	0	7	1
	0	2	1
	0	5	1
	1	8	2
	0	1	1
	4	1	4
	0	0	0
	1	2	2
	3		3
	3	25	3
	2	12	1

1	0	0
1	22	3
8	2	4
1	2	2
4	20	2
1	15	3
4	48	3
2	7	2
1	9	1
1		1
4	5	4
1	5	1
1	17	1
3		3
0	7	1
0	1	0
1	20	1
2	11	2
2	47	
5	19	1
3	6	0
0	22	0
1	1	1
0	0	0
3	3	1
0	0	1
0	0	0
0	0	0
0	0	0
0	0	0
0	7	0
0	5	0
0	13	0
0	24	0
0	1	0
0	0	0
0	1	0
0	1	0
1	0	0
1	2	1
0	3	0
0	5	0
0	0	0
0	0	0
0	2	0
0	4	0
0	8	0
0	0	0
0	0	0
0	0	0
0	0	0
0	5	0
2	11	0
0	3	0
4	9	0
0	4	0
0	4	0
0	3	0

0	11	0
0	0	0
0	1	0
0	4	0
0	0	0
0	6	0
0	2	0
0	0	0
1	0	0
0	0	0
0	5	1
3	1	2
0	3	0
0		0
0	2	1
2	20	0
0	22	0
0	4	0
0	8	0
0		0
4	9	0
4	0	0
0	0	0
0	1	0
0	2	0
0	10	0
0	1	0
0	14	2
0	0	0
0	1	0
0	4	0
0	22	0
0	0	0
0	0	0
0	0	0
0	0	0
0	0	0
0	0	0
5	8	2
0	0	0
0	3	0
0	1	0
0	0	0
0	0	0
0	22	0
0	0	0
0	0	0
0	12	0
0	0	0
0	0	0
0	1	0
0	6	0
0	3	0
0	33	0
0	0	0
0	0	0
0	1	0
0	0	0

0	0	0
0	0	0
0	0	0
0	0	0
0	24	0
0	8	1

MGFA-PIS (CSR:0-E:6)	the use of oral steroids	dose of pyridostigmine bromide
6	1	180
6	1	30
6	1	90
6	1	0
6	1	180
6	1	180
6	1	180
5	1	180
5	1	180
5	1	180
5	0	30
5	1	180
5	0	90
5	0	120
5	1	60
5	0	120
4	0	180
4	0	60
4	0	60
4	0	0
4	1	120
4	0	15
4	0	180
4	1	120
4	0	120
4	0	60
4	0	180
4	1	60
4	0	240
4	0	240
4	0	30
4	1	180
4	0	120
4	1	60
4	1	60
4	1	120
4	1	60
4	0	120
4	1	0
4	0	120
4	1	0
4	1	0
4	1	180
4	0	0
4	0	180
4	0	0
4	0	
4	1	240
4	1	180
4	0	180
4	1	180
4	1	180
4	1	180
4	1	120

4	1	180
4	0	0
4	1	180
4	1	180
4	0	180
4	1	0
4	1	180
4	0	180
4	1	0
4	1	60
4	1	0
4	1	0
4	1	0
4	0	120
4	1	60
4	1	360
4	1	120
4	1	180
4	0	180
4	0	120
4	0	180
4	1	0
4	0	240
4	1	240
4	1	180
4	0	0
4	0	240
4	1	180
4	1	0
4	1	0
4	1	180
4	0	120
3	0	120
3	1	180
3	1	180
3	1	180
3	1	0
3	1	0
3	1	180
3	1	0
3	0	90
3	1	180
3	0	180
3	1	180
3	1	180
3	0	240
3	1	180
3	1	60
3	1	120
3	1	180
3	1	120
3	1	180
3	1	120
3	1	120
3	1	0
3	1	180
3	1	180
3	1	60

3	1	180
3	1	120
3	0	30
3	1	120
3	1	60
3	0	180
3	0	120
3	0	60
3	1	120
3	1	120
3	1	180
3	1	180
3	1	180
3	1	180
3	1	120
3	0	60
3	1	120
3	1	0
3	1	0
3	1	0
3	1	0
3	1	0
3	1	0
3	1	0
3	1	0
3	1	0
3	1	0
3	1	60
3	1	60
3	1	0
3	1	120
3	1	0
3	1	120
3	1	120
3	1	0
3	1	120
3	1	0
3	1	0
3	1	180
3	1	60
3	1	0
3	1	180
3	1	120
3	1	0
3	1	0
3	1	60
3	1	120
3	1	120
3	1	60
3	1	180
3	1	180
3	0	180
3	1	120
3	1	60
3	0	180
3	1	60
3	1	240
3	1	120

3	1	0
3	0	
3	0	180
3	1	180
3	1	60
3	1	180
3	0	
3	1	60
3	1	0
3	1	180
3	1	120
3	1	120
3	1	180
3	0	180
3	0	60
3	1	0
3	1	60
3	1	180
3	1	180
3	1	60
3	0	120
3	0	180
3	1	180
3	0	180
3	1	
3	1	0
3	1	0
3	1	0
3	0	180
3	1	180
3	1	180
3	1	0
3	1	360
3	0	180
3	1	180
3	1	0
3	1	0
3	1	180
3	1	180
3	1	180
3	1	180
3	1	180
3	1	180
3	0	180
3	1	180
3	1	60
3	1	120
3	1	120
3	1	0
3	1	60
3	1	0
3	1	0
3	0	120
3	1	0
3	1	180
3	1	120
3	0	120
3	1	120

3	1	120
3	0	360
3	1	180
3	1	0
3	0	0
3	1	0
3	0	180
3	1	180
3	1	180
3	1	60
3	1	0
3	1	120
3	1	0
3	1	120
3	1	0
3	1	120
3	0	120
3	1	0
3	0	180
3	0	120
3	1	120
3	1	0
3	1	60
3	1	0
3	1	180
3	0	120
3	1	0
3	1	0
3	1	240
3	1	60
3	1	240
3	1	240
3	1	180
3	1	60
3	1	0
3	1	120
3	1	240
3	1	0
3	1	60
3	1	120
3	1	180
3	1	180
3	1	180
3	1	120
3	1	0
3	1	180
3	1	120
3	1	180
3	1	180
3	1	0
3	1	180
3	1	180
3	0	0
3	1	0
3	1	0
3	1	0
3	1	180
3	1	0

3	1	0
3	1	180
3	0	180
3	1	0
3	1	120
3	1	240
3	1	120
3	0	180
3	1	240
3	1	180
3	0	180
3	0	120
3	1	120
3	1	360
3	1	120
3	1	120
3	1	180
3	0	60
3	1	0
3	1	120
3	1	0
3	1	0
3	0	180
3	1	180
3	1	120
3	1	90
3	1	240
3	1	180
3	1	240
3	0	60
3	1	180
2	1	180
2	1	0
2	1	60
2	0	120
2	0	60
2	1	180
2	1	180
2	1	15
2	1	10
2	0	
2	1	15
2	1	120
2	1	120
2	1	0
2	0	0
2	1	180
2	0	60
2	1	0
2	0	30
2	1	180
2	0	120
2	0	0
2	0	120
2	0	30
2	1	60
2	1	60
2	1	0

2	0	120
2	0	60
2	0	180
2	1	180
2	1	0
2	0	60
2	0	0
2	1	120
2	0	30
2	1	0
2	1	0
2	0	0
2	1	60
2	0	120
2	1	180
2	0	120
2	1	120
2	1	180
2	1	30
2	1	120
2	1	0
2	1	0
2	1	0
2	1	0
2	1	120
2	1	0
2	1	0
2	1	0
2	1	120
2	1	0
2	0	90
2	1	0
2	1	60
2	1	120
2	1	0
2	1	0
2	1	0
2	1	180
2	0	120
2	1	0
2	1	0
2	1	0
2	1	60
2	1	0
2	0	120
2	1	180
2	1	60
2	1	120
2	1	0
2	1	120
2	1	0
2	1	0
2	0	60
2	0	120
2	1	180
2	1	0
2	1	0
2	1	0

2	1	120
2	1	0
2	0	120
2	0	90
2	0	180
2	1	180
2	1	0
2	1	0
2	1	0
2	1	120
2	1	0
2	1	60
2	0	180
2	0	120
2	1	0
2	1	60
2	0	60
2	0	180
2	1	60
2	0	0
2	1	0
2	1	0
2	0	120
2	1	0
2	0	60
2	0	120
2	1	15
2	1	120
2	0	120
2	0	120
2	1	120
2	1	180
2	1	120
2	1	180
2	0	60
2	1	120
2	0	120
2	1	120
2	0	180
2	0	120
2	1	180
2	1	180
2	0	180
2	1	180
2	0	60
2	1	90
2	0	60
2	1	180
2	1	240
2	0	60
2	1	180
2	0	180
2	1	0
2	0	180
2	1	180
2	0	180
2	1	60
2	0	180

2	1	120
2	1	30
2	0	180
2	1	180
2	1	0
2	1	180
2	1	120
2	1	180
2	1	120
2	1	120
2	0	180
2	1	120
2	1	60
2	1	90
2	0	60
2	1	0
2	1	180
2	1	120
2	1	60
2	0	60
2	0	60
2	1	0
2	1	0
2	1	0
2	1	0
2	1	0
2	0	0
2	1	60
2	1	120
2	1	120
2	1	0
2	0	120
2	0	120
2	1	120
2	1	120
2	1	180
2	1	60
2	1	60
2	0	0
2	1	240
2	1	60
2	0	120
2	1	180
2	1	0
2	0	60
2	0	180
2	0	0
2	0	0
2	0	120
2	1	0
2	1	0
2	1	0
2	0	180
2	1	0
2	0	60
2	0	120
2	0	0
2	1	180

2	1	120
2	1	0
2	0	0
2	0	0
2	1	60
2	0	120
2	0	180
2	1	60
2	0	0
2	0	60
2	0	180
2	1	180
2	1	0
2	1	0
2	1	180
2	0	120
2	0	60
2	0	60
2	1	60
2	1	120
2	1	90
2	1	0
2	1	0
2	0	180
2	1	180
1	1	60
1	0	60
1	1	0
1	1	0
1	1	0
1	1	0
1	1	0
1	1	0
1	1	0
1	1	0
1	1	0
1	1	0
1	1	0
1	1	0
1	1	0
1	1	0
1	1	0
1	1	0
1	1	0
1	1	0
1	1	0
1	1	0
1	1	0
1	1	0
1	1	0
1	1	0
1	1	0
1	0	0
1	1	0
1	1	0
1	1	0
1	1	0
1	1	0

1	1	0
1	1	0
1	0	0
1	0	0
1	1	0
1	1	0
1	1	0
1	1	0
1	1	0
1	1	0
1	1	0
1	1	120
1	1	0
1	1	0
1	1	120
1	1	0
1	1	0
1	1	0
1	1	0
1	1	0
1	1	0
1	1	0
1	1	0
1	1	0
1	1	0
1	1	0
1	1	0
0	0	30
0	1	180
0	0	0
0	1	0
0	0	0
0	0	0
0	0	0
0	0	0
0	0	0
0	1	0
0	1	0
0	0	0
0	1	0
0	1	0
0	1	0
0	0	0
0	1	0
0	1	0
0	1	0
0	1	0
0	1	0
0	1	0
0	1	0
0	0	0
0	0	0
0	1	0
0	1	0
0	1	0
0	1	0
0	1	0
0	0	0

0	0	0
0	0	0
0	0	0
0	0	0
0	1	0
0	0	0
